# The ethics of blood stewardship and quantitative futility assessments to predict certain death in neurotrauma patients

**DOI:** 10.3389/fmed.2026.1730579

**Published:** 2026-04-21

**Authors:** Marie N. Karam, Jenny Chen, Samuel J. Thomas, Miguel Velasco, Afsheen Mansoori, Molly L. Feller, Mark D. Fox, Ernest E. Moore, Hunter B. Moore, Peter K. Moore, Vraj S. Patel, Jeffery M. Bao, Connor P. Schmitt, Joseph G. Robuck, Aleksey T. Zielinski, Scott G. Thomas, Daniel F. Lewandowski, Connor M. Bunch, Futile Indicators for Stopping Transfusion in Trauma (FISTT) Collaborative Group

**Affiliations:** 1 Indiana University School of Medicine—South Bend Campus, South Bend, IN, United States; 2 Department of Emergency Medicine, Saint Joseph Mishawaka Medical Center, Mishawaka, IN, United States; 3 Department of Surgery, Ernest E. Moore Shock Trauma Center, Denver Health, Denver, CO, United States; 4 Department of Surgery, University of Colorado School of Medicine—Anschutz Medical Campus, Aurora, CO, United States; 5 AdventHealth Transplant Institute Porter, Denver, CO, United States; 6 Department of Medicine, University of Colorado School of Medicine—Anschutz Medical Campus, Aurora, CO, United States; 7 Royal College of Surgeons in Ireland, Dublin, Ireland; 8 Beacon Medical Group Trauma & Surgical Research Services, South Bend, IN, United States; 9 Department of Emergency Medicine, Henry Ford Hospital, Detroit, MI, United States

**Keywords:** blood component transfusion, emergency medicine, hemorrhage, medical ethics, medical futility, resuscitation, trauma, traumatic brain injuries

## Abstract

Universally accepted guidelines to predict futile resuscitation in severely bleeding trauma patients with traumatic brain injury do not exist. These patients may consume vast volumes of blood products in futile cases, which is especially problematic during times of local and national blood scarcity. However, determining which patients have no chance of survival is complicated and often reliant on the traumatologist’s individual judgment, which may be inconsistent. Traumatologists often face the ethical dilemma of balancing their obligations to provide appropriate care for patients and to conserve blood products for other patients. To assist physicians, bedside futility algorithms have been developed, some of which emphasize the negative effects of traumatic brain injury on survival. Bedside futility algorithms may be used during futility time-outs early in the treatment of severely bleeding trauma patients who are unlikely to survive, potentially preventing blood product waste by providing guidance to clinicians in the early determination of futility and the withdrawal of life-sustaining treatment. These algorithms are steps toward the development of ethically grounded, data-driven clinical guidelines regarding the use of blood products in severely bleeding trauma patients. We compare historical and nascently proposed futility algorithms in the context of the ethical challenges of declaring futility in the severely injured population.

## 1 Introduction

Despite decades of research concerning the withdrawal of life-sustaining treatment (WLST) for severely bleeding trauma patients (SBTPs), including those with traumatic brain injury (TBI) with or without associated extracranial hemorrhagic shock, there remains a lack of consensus among critical care physicians and their colleagues on formulating specific protocols for WLST (1–11). Recently, it has been noted that administrative guidelines, which attempt to address the different ethical problems and medical legalities in patient care, have not produced evidence unanimously accepted by critical care medicine and trauma groups (4). In an attempt to address this lack of universally accepted guidelines for WLST for SBTPs with TBI, international bodies related to critical care have made recommendations to provide a comprehensive system that can aid practitioners in the process of transitioning these patients who consume great amounts of scarce medical resources to comfort care in order to minimize ineffective treatments and preserve resources, while avoiding the trap of the self-fulfilling prophecy inherent in the decision-making process of WLST (1, 4, 8, 12–22).

The lack of homogeneity among institutions and clinicians for defining futility carries increased significance given the recent local and national blood shortages in the United States, particularly with traumatologists’ use of both balanced hemostatic resuscitation of 1:1:1 packed red blood cells (PRBCs):fresh frozen plasma (FFP):platelets and whole blood resuscitation as massive transfusion (MT) strategies for SBTPs with or without TBI (5, 11, 23–32). Evidence for the efficacy of early 1:1:1 hemostatic resuscitation was established by the 2015 Pragmatic, Randomized Optimal Platelet and Plasma Ratios (PROPPR) trial, which was a multicenter randomized controlled trial that compared administration of MT with 1:1:1 to 2:1:1 PRBCs:FFP:platelets in SBTPs. The patients in the 1:1:1 group were more likely to achieve improved hemostasis and had fewer deaths from exsanguination in the first 24 h. However, the primary outcomes of 24-h and 30-day mortality were not statistically different between the two groups (32). Since the PROPPR trial’s publication, 1:1:1 balanced hemostatic resuscitation has become the standard of care in MT for SBTPs, leading to an increased rate of blood component use in trauma (5). Additionally, the COVID-19 pandemic has caused a lasting reduction in the nation’s blood supply due to the reduced number of donation opportunities and the lower number of blood donations as a result (33–41). The increase in trauma cases and healthcare use after the pandemic has significantly depleted resources (34). This shortfall of blood products, combined with increased usage, has prompted the formulation and publication of algorithms based on bedside parameters intended to reliably predict early death for SBTPs with or without TBI (2, 5–7, 11, 23, 25, 41).

Trauma scores have been used to triage trauma patients, predict patient outcomes, guide patient expectations, and form a foundation for quality assessment (42). While there have been various attempts to define futility at the bedside for SBTPs, the majority of these scores, such as the Sequential Organ Failure Assessment (SOFA), Acute Physiology and Chronic Health Evaluation II (APACHE II), Abbreviated Injury Scale (AIS), Injury Severity Score (ISS), and Trauma and Injury Severity Score (TRISS), are not easily applied early in resuscitation because the data required to accurately calculate these scores cannot be easily collected within the first few hours by the traumatologist (2, 34, 35, 42–51). Many of these scores, along with those that are quick to obtain, such as the Revised Trauma Score, are still inaccurate in predicting early death for SBTPs (52–54). Despite the many types of scoring systems, there are none that are universally accepted and certainly none that have been applied to predict death within the first 4 h following severe trauma. These scoring systems have been demonstrated to be cumbersome and require long periods of observation for proper prognostication, and they are more useful for retrospective studies rather than point-of-care declarations of futility at the bedside (42, 53, 55, 56).

In the past, specific guidelines have attempted to give traumatologists direction for withholding or terminating resuscitation in the early prehospital and hospital environment for patients with injuries that will clearly not allow survival (57, 58). Algorithms have been based on large studies that did not consider the presence or absence of TBI in SBTPs, and others have failed to integrate the importance of severe TBI (sTBI) and age in determining certain death for SBTPs (2, 5, 6, 11). The purpose of this review is to address the recent attempts to define specific parameters for futility of continued resuscitation for SBTPs with or without sTBI and to propose an operational model able to define and indicate futile resuscitation (FR) in the early stages of care. Due to the lack of consistency in determining parameters to define patients with sTBI, particularly for those with concomitant severe extracranial injuries and severe hemorrhage for whom care should be withdrawn, there have been renewed attempts to revisit the ethical and practical aspects of defining specific parameters that can reliably predict certain death (1, 2, 6, 8, 11, 50).

## 2 Defining futility and futility criteria for trauma patients

Historically, clinicians and their colleagues, such as biomedical ethicists, have differed in both their definitions and applications of futility (3, 59, 60). The term “medical futility” has an inconsistent definition in the literature, which has led to the development of different categories of futility. While biomedical ethicists have suggested that the word “futile” should only be used in rare situations where a medical intervention cannot meet the intended patient outcomes, this has not prevented the term “futile” from being used in other ways with certain descriptors (3, 61). The term “qualitative futility” describes the value-laden quality-of-life judgment by the physician to declare futility, while “quantitative futility” aims to achieve decisions regarding futility based on probabilistic thresholds, such as 90% or 95%, for the likelihood of mortality with foundations in medical evidence, physiology, and clinical experience (3, 7, 61–66). This phenomenon of physicians relying on prior clinical experience to determine if and when futility should be declared has also been called clinical gestalt, a heuristic reliance on a series of previous experiences and patient interactions that each clinician believes to achieve a universal sense of certainty (67–72). Both quantitative and qualitative futility have been criticized for the uncertainty inherent in making decisions regarding patient care. While qualitative futility has been criticized for the fact that quality-of-life judgments would be made by the physician rather than the patient, quantitative futility has been criticized for having inconsistent and arbitrary probabilistic thresholds and for perhaps causing mortality after withdrawal of care rather than predicting it (a self-fulfilling prophecy) (3, 7, 8, 14, 16, 21, 66, 73–75).

On the other hand, the term “physiological futility” refers to procedures that have a 0% chance of meeting intended outcomes and is not based on individual evaluation by the physician but rather on the impossibility of the treatment having the desired effect for any such case (73). This definition is similar to the narrow definition of futility brought about by biomedical ethicists, since it reflects the rarity of a situation where the physician can be absolutely certain that a procedure is futile (3, 61, 73).

Futility-adjacent descriptors have also been coined to describe procedures that are not likely to meet the intended outcomes but are not definitively futile. Different terms have been used to describe this in-between status, such as “medically ineffective” or “potentially inappropriate” (3, 73). These terms reflect treatments that are unlikely to save the patient but might improve the patient’s state to a lesser extent or for a short period of time.

### 2.1 Quantitative futility criteria

Traumatologists have recently attempted to refine the assessment of quantitative futility by using probabilistic algorithms to define a transfusion cut-point for futility. However, this has predictably resulted in controversy (2, 5–7, 11, 23, 25, 76, 77). Given the complexity of the pathophysiologic and anatomic derangements that occur following trauma, attempts to define quantitative futility can only approach the certainty that patients will die and always involve a qualitative aspect. Because defining a transfusion cut-point for FR is challenging, these studies have attempted to approach each complex decision to withdraw care for SBTPs using actively evolving decision tools (2, 5, 7, 11, 23, 25, 63, 64, 76, 77). These decision tools will form the foundation for future studies on futility and allow traumatologists to improve futility predictions for SBTPs with or without TBI.

For patients with TBI with or without extracranial hemorrhage, there have been advancements in predicting death within 24 h of admission and at different intervals pre- and post-discharge in the form of predictive scoring systems. The Focused Outcomes Research in Emergency Care in Acute respiratory distress syndrome, Sepsis, and Trauma (FORECAST) group has shown that TBI and high disseminated intravascular coagulation FORECAST scores for patients immediately upon arrival at the hospital were highly predictive of in-hospital mortality (78). Upon further development, such algorithms may facilitate faster and more accurate triaging of patients, especially geriatric patients with TBI, to palliative care. The quick Elderly Mortality After Trauma (qEMAT) score may be used to quickly predict in-hospital mortality upon admission and could possibly assist in predicting futility (79). Other prediction tools include the Prehospital Injury Mortality Score; shock index; prehospital ABC score; Prehospital Mortality Prediction Score; Probability of Survival score; Trauma Rating Index in Age, Glasgow Coma Scale, Respiratory rate, and Systolic blood pressure score (TRIAGES); and the Commands, Age, Pulse rate, Systolic blood pressure, and peripheral Oxygen saturation (CAPSO) model (2, 42, 50, 51, 54, 80–84).

Among the many clinical, laboratory, and radiologic parameters that have been used to predict certain death for SBTPs with or without brain injuries, the use of transfusion cut-points per hour has been proposed to address the previously mentioned scarcity of blood products (23). However, these attempts have failed to determine cut-points based on transfusion volumes alone; therefore, the reliance on a combination of clinical and laboratory parameters, including transfusion cut-points per hour, has been proposed with the goal of reaching 100% positive predictive value (PPV) and specificity (5, 11, 23, 25). Achieving 100% PPV and specificity would allow traumatologists to define parameters that will reliably predict certain death for SBTPs. Specifically, PPV is defined as True Positives/(True Positives + False Positives), whereas specificity is defined by True Negatives/(True Negatives + False Positives). If a clinical predictor has no false positives, then the PPV and specificity are 100%. Combinations of parameters, such as severe hypotension, hyperfibrinolysis, high lactate, high international normalized ratio (INR), low pH, high base deficit, low Glasgow Coma Scale (GCS), and cardiac arrest with return of spontaneous circulation (ROSC), have been cited as highly accurate predictors of death (5, 11, 85).

These probabilistic parameters can be used to predict death for patients whose fatality within hours of arrival is inevitable. However, there remains a large group of patients who consume significant quantities of blood products during the early hours of resuscitation for whom no algorithms or guidelines exist to regulate continued resuscitation that most likely will end in fatality. A major limitation of the Suspension of Transfusion and Other Procedures (STOP) criteria, which define combinations of parameters with 100% PPV and specificity, is that the data are derived from the PROPPR trial. The study looked at the survival of trauma patients and their laboratory values. It was noted that up to 50% of the trauma patients in the datasets presented with TBI, but they were not distinguished from the non-TBI patients during analysis (2, 5, 6, 32, 86). Without this delineation, there is a lack of clarity regarding the specific resuscitation needs of those with TBI versus those without TBI (2, 6, 11, 32, 86–89). Previously, there have been attempts to accurately define futility for severely injured children and adolescents with or without sTBI based on bedside parameters (11, 85). This futility algorithm, which identifies Futility Cut-Off Points for Children and Adolescents (FCOPCA), demonstrates 100% PPV and specificity, and identifies patients who exhibit one of several combinations of bedside markers, like the STOP algorithm does. However, FCOPCA uses TBI (AIS_head_ ≥ 3) as a marker of futility, unlike the STOP criteria. This algorithm could allow traumatologists to efficiently declare futility in children and adolescents, an objective that has lacked definitive measures to assist clinicians (5, 11, 85, 90).

Deciding to stop resuscitative measures for severely injured older adults is also challenging for both clinicians and families. Historically, these decisions relied on clinical gestalt rather than evidence. Recently, a prediction tool called the Futility of Resuscitation Measure (FoRM) has been proposed with the intent of reliably predicting futility in severely injured older patients and lessening reliance on clinical gestalt (7). FoRM was developed based on trauma patients aged 60 years or older who received blood products within 4 h and did not undergo WLST. It relies on a point system, whereby a FoRM score greater than 20 results in a 95% chance of mortality. FoRM emphasizes the importance of age > 80 combined with frailty, the presence of TBI, systolic blood pressure (SBP) < 50 mmHg, the use of vasopressors, the administration of 20 U PRBCs in 4 h, and the presence of a midline shift, which would be associated with at least 95% mortality and are likely events for SBTPs with TBI. Even the presence of just TBI, age > 80, frailty, and midline shift before the administration of any blood products predicts a mortality rate of over 50% (7). Evidently, addressing the presence of TBI and age early in the assessment of futility for SBTPs represents an important first step that has not been highlighted until recently in the literature. The parameters for predicting futility with greater than 95% PPV from the FoRM criteria still do not achieve 100% PPV or specificity, unlike the STOP or FCOPCA criteria (Table 1). As we have seen for children and adolescents, the inclusion of TBI can be used to predict death (7, 11, 85, 91, 92).

**Table 1:** Summary of the suspension of transfusion and other procedures (STOP), futility cut-off points for children and adolescents (FCOPCA), and futility of resuscitation measure (FoRM) algorithms for predicting futility.

STOP Markers with 100% PPV and Specificity (> 15 years old)		
Arrival SBP ≤ 50 mmHg and Lactate ≥ 15 mmol/L	Arrival SBP ≤ 50 mmHg and LY30 ≥ 30%	Arrival SBP ≤ 70 mmHg, Lactate ≥ 15 mmol/L, and LY30 ≥ 30%
ROSC and LY30 ≥ 30%	ROSC and Lactate ≥ 12 mmol/L	ROSC and Field GCS = 3
FCOPCA Markers with 100% PPV and Specificity (< 16 years old)		
Arrival pH ≤ 7.00 and INR ≥ 2.0	Arrival Base Deficit ≥ 20 and INR ≥ 2.0	INR ≥ 2.0 and sTBI
Arrival Base Deficit ≥ 12 and LY30 ≥ 20%	Arrival pH ≤ 7.05 and LY30 ≥ 20%	LY30 ≥ 20% and sTBI
FoRM Markers (≥ 60 years old)		Points (Point Sum > 20 Associated with 95% Mortality)
Frailty; Resuscitative Endovascular Balloon Occlusion of the Aorta; TBI Midline Shift; Craniectomy		1
70–80 Years Old; Vasopressor Use Within 6 Hours		2
> 80 Years Old; 6–10 U PRBCs Transfused in 4 Hours		3
≥ 1 Episode of SBP < 50 mmHg; 11–15 U PRBCs Transfused in 4 Hours		6
Prehospital Cardiac Arrest; sTBI and GCS ≤ 8; 16–20 U PRBCs Transfused in 4 Hours		7
Emergency Department Thoracotomy; > 20 U PRBCs Transfused in 4 Hours		9

The three algorithms were developed for different age groups, and while STOP and FCOPCA propose markers that have 100% positive predictive value (PPV) and specificity, FoRM provides a scoring system that predicts the chance of futility at different total point sums (5, 7, 11, 85). Glasgow Coma Scale (GCS), international normalized ratio (INR), percent amplitude reduction of the clot 30 min after maximum amplitude (LY30), return of spontaneous circulation (ROSC), systolic blood pressure (SBP), severe traumatic brain injury (sTBI), units packed red blood cells (U PRBCs).

It can be seen when comparing the STOP and FoRM criteria that TBI, age, and the units of PRBCs per hour are significant prognostic indicators for certain death in the FoRM criteria but not STOP. This may indicate the importance of age in determining which markers are relevant, as FoRM was developed using elderly patients (≥ 60 years old), while STOP was developed with a database of patients > 15 years old (5, 7). Studies in children and adolescents have demonstrated that in the absence of TBI, transfusion cut-points that can predict futility are ill-defined (92). However, the presence of TBI significantly reduces the chance of survival, such that transfusion cut-points may be useful in predicting death in patients with extracranial injury who fail to respond to the efforts of resuscitation, as shown in FCOPCA (11, 85). A comparison of the characteristics of STOP, FCOPCA, and FoRM is presented in Table 2.

**Table 2:** Compares the significant characteristics of STOP, FCOPCA, and FoRM (5, 7, 11, 85).

Characteristic	STOP	FCOPCA	FoRM
100% PPV and Specificity	✓	✓	
Includes Graded Percent Chances of Death			✓
Includes Elderly Age as Marker			✓
Includes TBI as Marker		✓	✓
Includes Transfusion Volume of PRBCs as Marker			✓
Includes Lactate as Marker	✓		
Includes pH and/or Base Deficit as Marker		✓	
Include INR as Marker		✓	
Includes Viscoelastic Testing Data as Marker	✓	✓	
Includes ROSC as Marker	✓		✓

Different quantitative futility algorithms have found different significant predictors of futility for different age groups.

The presence of sTBI associated with extracranial hemorrhage, whether in adolescents or adults, also compounds the underlying trauma-induced coagulopathy. The addition of age as a parameter alongside the presence of sTBI with extracranial hemorrhage allows for a more physiologically rigorous tool to predict FR (2, 6, 93). Because age appears to be fairly reliable in predicting early futility, many studies have tried to quantify the effect of age on death in trauma patients, including scores such as the Geriatric Trauma Outcome Score, GERtality score, Trauma-Specific Frailty Index, APACHE II, TRISS, qEMAT, TRIAGES, and CAPSO (7, 11, 44, 47, 48, 51, 54, 79, 81, 83, 94–102). Even though the effect of age on mortality for trauma patients has been observed in the literature, futility algorithms still do not consistently incorporate geriatric age into their parameters (2, 5–7, 11, 26, 28, 29, 47, 48, 51, 94–101, 103–115).

Using the presence of geriatric age, TBI, and other parameters from the STOP or FoRM criteria may allow the traumatologist to conserve blood products by indicating earlier termination of resuscitation efforts in older SBTPs with sTBI, while continuing aggressive resuscitation efforts for younger patients with severe hemorrhage due to isolated penetrating injury and no presence of TBI. The literature has already anticipated these findings with the observation that younger patients with these conditions can survive even after massive blood product consumption, and in this group of patients, there are very few well-defined thresholds for limiting resuscitative efforts based on transfusion cut-points alone (2, 5, 6, 27, 76, 92, 108, 116).

### 2.2 The effect of the COVID-19 pandemic on the need for quantitative futility criteria in trauma

Historically, resource allocation in medicine has been a duty that falls under the purview of physicians, and in settings of more extreme resource scarcity, physicians are more ethically obligated to allocate resources in a just manner (117–121). This was demonstrated during the COVID-19 pandemic, when uncertain pathology, equipment and resource constraints, and a time-pressured environment necessitated making real-time bedside ethical decisions (121). At the height of the pandemic, physicians were forced to distribute healthcare resources (e.g., ventilators, hospital beds, staff) to ensure distributive justice (36, 119, 121). To ration care justly, the futility of providing care to each patient had to be determined; in other words, using the available medical evidence, patients who had the highest chances of survival and the lowest chances of long-term complications were prioritized when rationing limited resources (41, 121).

While the national blood supply was under duress during the COVID-19 pandemic, the use of balanced hemostatic resuscitation (1:1:1 PRBCs:FFP:platelets) in trauma resuscitation increased, continuing a trend that developed shortly before the COVID-19 pandemic, along with whole blood resuscitation regaining favor and being utilized by an increasing number of institutions (24, 28–32). Although this led to increased survival chances in patients who previously may have died, it also increased the units of blood products consumed by patients with non-survivable injuries. Similarly, the increased interventions for COVID-19 patients consumed significant blood products but resulted in improved patient outcomes and the survival of patients who would have otherwise died (11, 39, 117, 118, 120, 122, 123). However, as with traumatologists’ increased use of balanced hemostatic resuscitation and whole blood, many blood products and other resources were used on COVID-19 patients who ultimately died (123). These approximately simultaneous events, the increase in blood products used for trauma cases and the negative impacts of the COVID-19 pandemic on the nation’s blood supply, increased the need for traumatologists and critical care specialists to find ethical and probabilistic tools to guide resource utilization. It should be of interest that recent literature confirming the need for futility time-outs (FTOs), which are designated times to pause resuscitation and consider declaring futility based on reliable parameters in an effort to conserve blood products, coincides temporally with the COVID-19 pandemic (5, 6, 23, 27, 37, 41, 43). The relative lack of resources and blood products that followed the COVID-19 pandemic has directly led to the call for the ethical evaluation of WLST from SBTPs with or without TBI because of a similar lack of resources and blood products (11, 40).

## 3 The four principles of biomedical ethics in relation to FR

The concepts of beneficence, nonmaleficence, autonomy, and justice have been advanced as four foundational principles of biomedical ethics (121, 124–130). An analysis of each of these four principles as they apply to ethical decisions to be made for SBTPs with TBI in a time-pressured environment is presented.

### 3.1 Nonmaleficence and beneficence

The ethical principle of nonmaleficence refers to the objective of doing no harm, and it compels healthcare professionals to treat patients in a manner that does not result in injury, suffering, or pain (125, 126, 129). Correspondingly, the principle of beneficence compels healthcare professionals to act with the goal of benefiting the patient (125, 126). Both the principles of nonmaleficence and beneficence may be applied in circumstances where a plan of care may carry the risk of negative impact upon the patient; however, in these situations, the plan of care must carry a potential positive consequence upon the patient that outweighs the risk (127, 128).

Nonmaleficence and beneficence work in tandem to ensure the best possible outcome for patients and adherence to ethical standards. In situations where WLST is being considered for SBTPs with TBI, these principles are extremely important, since WLST for patients who may still survive could violate the principle of beneficence, while continuing to provide care for a patient when it is futile could violate the principle of nonmaleficence if construed as causing unnecessary harm (126). Because nonmaleficence and beneficence allow for the observation of ethical and beneficial care of patients, they form the foundation of biomedical ethics upon which the additional principles, autonomy and justice, can be considered.

### 3.2 Autonomy

Autonomy represents the right of patients to make their own informed decisions regarding their care, and it is the physician’s responsibility to ensure that patients can make these informed decisions when possible (125, 127–129, 131). Autonomy allows patients to maintain their right to seek treatment, refuse care, or seek alternative interventions (131, 132). However, autonomy has its limits. Adults with decision-making capacity generally have autonomy when it comes to decisions about their own treatment, but it is beyond the ethical bounds of autonomy for patients to demand something at the expense of other patients (128). Likewise, patients with decision-making capacity have the right to refuse care, but in emergent circumstances without prior guidance for a patient’s directives regarding life-sustaining care, such as a do not resuscitate order, an emergency presumption of consent allows a medical professional to provide necessary care (126, 131, 132).

Cardiopulmonary resuscitation falls under the purview of this presumption of consent in emergent circumstances, absent a contrary directive from the patient. However, there has been considerable discussion on whether physicians should be able to withhold resuscitation from those patients whom they believe will not survive (4, 133). This proposal has received criticism, since these unilateral decisions made by physicians would disregard the value of the documented wishes of a patient (4, 134, 135). Objections such as these imply that determining futility should include value-laden judgments, so patients should have a say in their treatments. The conflict between the desire to respect patient wishes and the evolving parameters of futility rests at the heart of this ethical decision regarding autonomy and the traumatologist’s duty to treat dying trauma patients (134, 135). A discussion focused on the unique aspects of the ethical foundations of applying and abiding by the principle of autonomy in a time-pressured environment, such as SBTPs with TBI in the earliest moments of trauma resuscitation, is best described with this conflict between the universality of the application of autonomy and the need for individual application of reliable predictors of futility.

Unlike intensive care cases, where patients have been observed for extended periods and either the patient or the patient’s surrogate can render decisions regarding WLST, the application of autonomy during early resuscitation of patients with sTBI is rendered more difficult because the patients often do not have the ability or a surrogate to provide consent. Often in this situation, the patients will be in extremis and thus unable to express their values, expectations, or wishes. Due to these circumstances, the ethical basis for providing care for unresponsive patients, such as unconscious trauma patients, has been the best interests standard, where substituted judgment of what a typical individual would find beneficial is used (126, 136). If a patient or patient’s surrogate has reasonably requested resuscitation or blood product transfusion to be withheld, that request should be honored, as long as the request is free from coercion and mental incapacity due to injury (62, 137–139). In the situation where a physician believes that further resuscitation would be futile and the patient’s surrogate requests that resuscitation be continued, the surrogate’s wishes should not be unilaterally overridden by the physician, since there are no universal guidelines that guarantee futility for SBTPs (60). Consulting a third party, such as an ethics committee, may assist in decision-making by considering multiple perspectives and ensuring equitable care (1, 41, 140, 141).

Due to patients presenting with varying levels of consciousness and mental states, the concept of a patient’s autonomy is complex in the trauma environment. This is further complicated by the fact that there are no alternative interventions to resuscitation. These complications of patient autonomy solidify the concept of justice as foundational for making ethical decisions in time-pressured environments.

### 3.3 Justice

Justice has been defined as the fair and equitable treatment of patients (125, 129). All patients’ lives are of equal value and dignity, and they should not be treated differently based on characteristics irrelevant to the care they deserve. Distributive justice is the principle of justice that dictates how scarce healthcare resources are to be deployed to communities or patients (125, 129). In making these decisions, physicians must consider the needs of their current patient, other patients, and future patients (142).

Therefore, when deciding whether to withhold blood products from a moribund SBTP with TBI, a traumatologist can apply the concept of justice by asking, “Is it fair to continue transfusing a patient who has little chance of survival and consumes many blood products, which could deprive other patients who have better prognoses and are also owed resuscitation efforts?” As discussed previously, the scarcity of blood products has been exacerbated by traumatologists’ decisions to transfuse increasing amounts of blood, which are less likely to be replenished (5, 23, 25). Gestalt-based decisions on when to stop transfusion are accordingly liable to beget poor outcomes for both patients and blood product inventories (2, 5, 6, 123, 143). Guidelines that adhere to distributive justice should thus be established to regulate the usage of blood products in SBTPs with TBI, especially since ulterior motives, such as the lack of insurance in patients with isolated sTBI, have been found to be associated with early WLST, even though all patients deserve equitable care regardless of background and ability to pay (1, 74).

An important case for distributive justice in blood transfusion protocols can be made by way of analogy to the current paradigm of organ transplantation. Unlike with blood transfusions, physicians who deal with organ transplantation treat organs as a scarce resource, both in gross number available and timeliness of acquisition (144–146). Therefore, more sophisticated methods for situations regarding the provision of organs for deserving patients have been devised to determine which patients should receive organs and when. These devised guidelines, such as the Model for End-stage Liver Disease score for liver transplantations, systematize patient recipients to avoid *ad hoc* determinations by individual physicians and find the most severely ill patients who would benefit the most from transplantation (147). It should be noted that exclusionary criteria play an important role in justly utilizing scarce organ transplantation resources, since organ transplant wait lists consider aspects of patient medical history and predicted life expectancy to determine who will and who will not receive organ transplants (145, 146, 148, 149). The comparison between the transplantation of scarce organs and the use of scarce blood products for SBTPs who will most likely die regardless of continued transfusion of blood products during MT can be proposed as a foundation for creating specific guidelines that can be used at the bedside of SBTPs. While both blood and organs are considered to be living tissue, organ transplantation often requires eligibility criteria and a wait list, since organs are a scarce resource (145, 146, 148, 149). Blood is also a scarce resource, yet the rationing strategies for blood products are less defined (2, 143). Therefore, traumatologists should have similar strategies to identify which criteria warrant a patient’s exclusion from further transfusion.

It is also important to note that highly reliable markers that predict futility could help identify patients who would be viable candidates for WLST and subsequent controlled or uncontrolled donation after circulatory death (DCD) in the intensive care unit or emergency department, and patients who experienced prehospital cardiac arrest with unsuccessful resuscitation may be candidates for uncontrolled DCD (150–155). Futility criteria that include prehospital cardiac arrest or ROSC may guide traumatologists when declaring futility and help to preserve blood products for this group of patients (5, 7).

The analogy between organ donation and blood transfusion for SBTPs is useful but limited, since for organ transplant, selection committees most often have the time to thoroughly consider which patients will receive organs (149). However, in the acute first moments for SBTPs who are not responding to heroic resuscitation, traumatologists must make quick decisions on how to allocate blood, which can lead to rushed decisions. Additionally, blood, unlike organs, can be more easily obtained, and for this reason, patients and their families may not understand that blood products are a finite resource. This can create a conflict between societal expectations and the best course of care. Therefore, it is necessary to develop futility markers with 100% PPV and specificity that can be used in the time-pressured moments of resuscitation for SBTPs.

## 4 Application of ethics to developing bedside markers of futility for SBTPs with or without brain injury

We have reviewed the basic ethical principles of defining futility for SBTPs with the concepts of autonomy, justice, beneficence, and nonmaleficence. These principles are applied in the setting of the time-pressured environment of immediate care for these patients, and the presence of TBI serves as an important factor in predicting futility (2, 7, 11, 85).

An American College of Surgeons-Trauma Quality Improvement Program study of United States trauma centers has revealed that WLST occurs in only about 2–3% of trauma cases, but another study found that over 20% of sTBI patients underwent WLST (1, 10). Key independent markers for the indications for WLST were old age, low GCS, and sTBI (10). For time-pressured decision-making, there could be an incorporation of a futility checklist that includes these pertinent markers to reduce uncertainty. The importance of adhering to parameters that have 100% PPV and specificity is so that there can be no false positives for an algorithm that predicts certain death, since false positives would lead to the withdrawal of resuscitative efforts and could cause death instead of predicting it. To avoid false positives, no patients with injuries that have been identified as non-survivable should survive if resuscitative measures were to continue. Therefore, the search for the ideal clinical and laboratory parameters has sought to find a set of parameters that have a PPV and specificity of 100% and can be applied in the time-pressured environment of the trauma bay (2, 3, 5, 6, 11, 85).

### 4.1 From clinical gestalt to quantitative certainty

**Figure 1:**
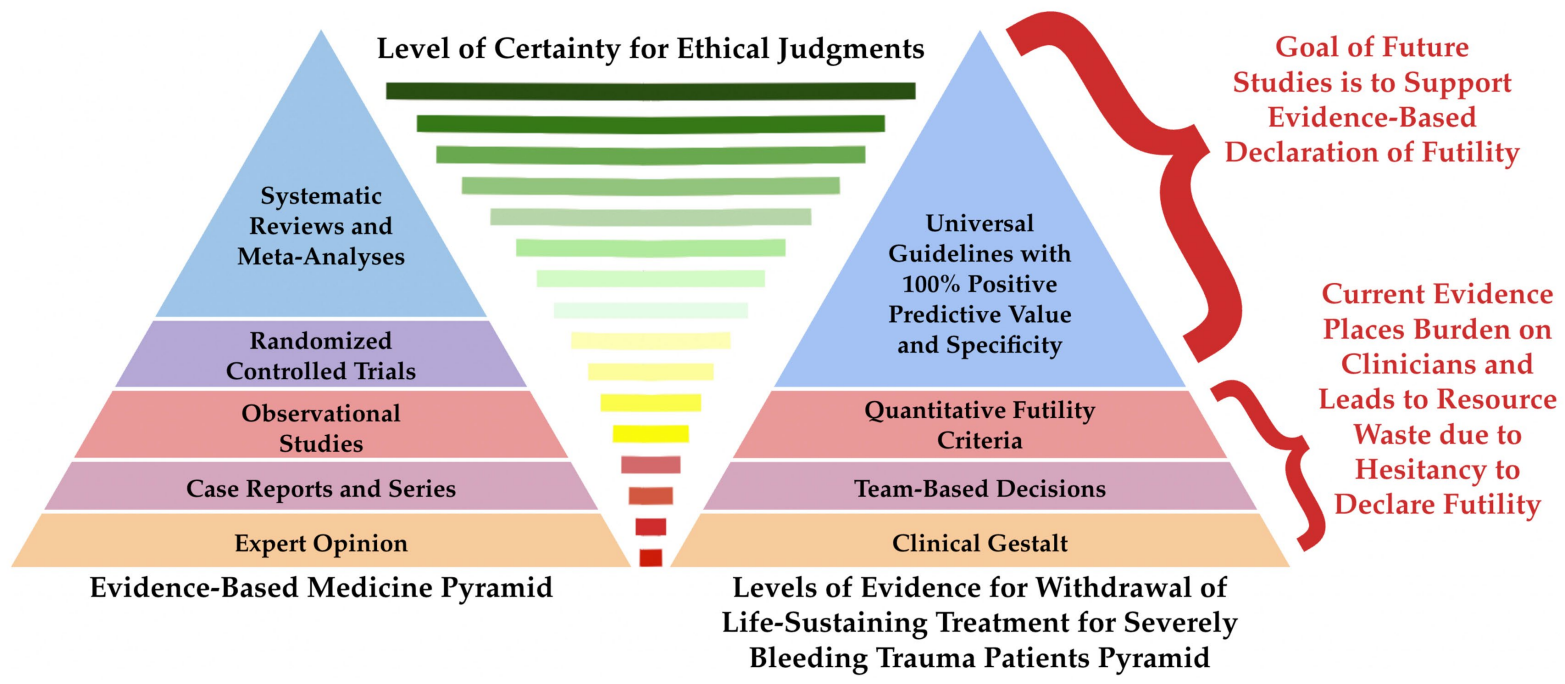
An analogy can be made between the decision to terminate resuscitation for severely bleeding trauma patients (SBTPs) with TBI (right) and the evidence-based medicine pyramid (left), from an individual expert opinion to systematic reviews and meta-analyses (157–159). Ethical principles inform both pyramids of evidence at every tier, but there are higher levels of certainty in making ethical judgments at the higher tiers of both pyramids. Though the current literature regarding establishing evidence-based guidelines that allow for the prediction of certain death for SBTPs is still in its infancy, as most algorithms and futility criteria are based on observational studies, it is anticipated that future randomized controlled trials, systematic reviews, and meta-analyses can be applied to one day create universal guidelines with 100% positive predictive value and specificity.

Recent attempts to quantify futility, as exemplified by the STOP, FCOPCA, and FoRM criteria, are based on an ethical construct with a goal of defining parameters for futility for SBTPs with or without TBI who fail resuscitative efforts. Previous tools designed to assist the clinician in withdrawing care for severely injured or ill patients have not been done in a time-pressured environment, since they rely on prediction scores, such as SOFA, APACHE II, AIS, ISS, TRISS, and others, that can require several hours to days of evaluation to calculate accurately (2, 6, 11, 33–35, 43, 45, 47, 51, 77, 103, 110, 156). In the time-pressured environment of trauma, a pyramid of certainty can be proposed, with the tip of the pyramid representing the current goal of guidelines with 100% PPV and specificity. This pyramid, showing the levels of evidence for WLST for SBTPs, can be compared to the well-known evidence-based medicine (EBM) pyramid (157–159). Figure 1 describes the evolution based on an ethical framework from a physician’s clinical gestalt to the elusive guidelines that have 100% PPV and specificity for certain death in SBTPs with or without TBI (2, 5–7, 11, 49, 72, 85).

Traditionally, evidence based on clinical cases or hypothesis-generating medical information remains at the bottom of the EBM pyramid and is the basis for expert opinion (160). This expert opinion on the EBM pyramid can be compared to clinical gestalt in the levels of evidence for WLST for SBTPs pyramid, as they are both based on opinion and experience rather than quantitative algorithms or other evidence. Physicians have relied on clinical gestalt to predict which patients would require MT for decades (72, 161, 162). Traumatologists have also used clinical gestalt to determine which patients will not survive trauma resuscitation, permitting traumatologists to make decisions about futility and blood product allocation (44, 67, 68, 72).

The comparison of the EBM pyramid to the levels of evidence for WLST for SBTPs pyramid from gestalt decision-making to universal guidelines with 100% PPV and specificity is useful, as each ascending level of the pyramids represents an increasing level of certainty. The recent search for parameters that can reliably predict futility for SBTPs reflects the urgency of defining futility for these patients. One cause of this urgency is the lack of blood products and the increased use of blood products by traumatologists with the use of balanced hemostatic resuscitation and whole blood, which has spurred inquiry regarding the difficult decisions regarding end-of-life medical care. A solution to this inquiry will not be simple, since despite more than 50 years of advanced resuscitation protocols in use, discussion in the medical literature and in the public arena is still characterized by controversy and lack of clarity concerning end-of-life decisions, whether related to traumatic or non-traumatic diseases (2, 5, 6, 11, 135).

In summary, the physician’s traditional response to shifting ethical standards and uncertainty regarding prognosis has been to rely on clinical gestalt (2, 67, 68, 72). However, the literature has shown that there is significant heterogeneity regarding the application of these gestalt-based decisions when applied to WLST for patients with TBI (1, 2, 12). As a response to this incongruent and heterogeneous use of clinical gestalt to predict futility for SBTPs, quantitative futility criteria that approach but never arrive at certainty have been proposed. The gap between these metric-assisted parameters, which closely predict futility, and certainty is filled by the clinical gestalt of the practitioner (7, 67, 68, 72). Recent shortages of blood products and consumption of healthcare resources for young and old SBTPs have resulted in the publication of decision rules that aim for certainty in developing clinical and laboratory parameters with 100% PPV and specificity (2, 5–7, 11, 23, 25, 41). It is within this framework that a specific identification of those clinical and laboratory parameters can now be attempted.

## 5 Proposal for defining a futility timeline for SBTPs with TBI based on ethically informed guidelines

Having framed the discussion regarding the determination of futility for SBTPs within the context of a pyramid of increasing certainty culminating in guidelines that have 100% PPV and specificity, it is now possible to propose the use of specific parameters that have a high rate of accuracy in predicting certain death in this group of patients. To better understand futility measures for TBI patients, it is useful to first discuss different categories of TBI in relation to their consumption of blood products during resuscitation and the effect of the hemorrhagic coagulopathy of TBI on the prognosis of patients.

### 5.1 Defining TBI categories in relation to early declaration of futility

For the purposes of defining the groups of TBI patients with and without extracranial hemorrhage, we propose a spectrum of categories of severity from isolated TBI (iTBI) to patients with sTBI and other injuries. First, iTBI is defined as TBI without extracranial injuries or coagulopathy. Therefore, iTBI patients do not require blood products for resuscitation. Second, hemorrhagic isolated TBI (hiTBI) is defined as TBI without extracranial injuries but with coagulopathy of TBI. Patients with hiTBI rarely require MT, depending on the definition of MT. However, they often consume blood products within the first 4 h, and the presence of coagulopathy makes them candidates for early declaration of futility depending on the presence of other parameters associated with futile resuscitation (163–169). Third, polytraumatic TBI (ptTBI) is defined as TBI with both extracranial injuries and hemorrhagic shock. Polytraumatic injuries are caused by traumatic injuries to both the brain and extracranial structures, and ptTBI patients also require MT. This spectrum is supported in the literature and defined by high AIS_head_ scores and the presence or absence of extracranial injury and coagulopathy (2, 13, 14, 16, 48, 56, 78, 93, 170–177).

### 5.2 Proposed resuscitation timeline for FTOs

It has been proposed in the literature that FTOs may be called to consider the declaration of futility for SBTPs and to conserve blood products (5, 6, 23, 27, 37, 41, 43). Traditionally, it is standard protocol for traumatologists to wait at least 72 h before considering futility for patients who have iTBI, although this guideline has been shown to not be uniformly applied (8, 13–15). For hiTBI patients, consideration of futility may occur at variable times since these patients have variable presentations, but futility may be considered before 72 h. For ptTBI patients, the likelihood of futility should be considered during the first 4 h of resuscitation, since many of these patients in particular are likely to die early and consume high volumes of blood products (2, 5, 16, 23, 25, 33–35, 37, 43, 93, 177–179).

We emphasize the utility of highly predictive guidelines for WLST for those patients with TBI associated with hemorrhage in a time-pressured environment, which allows for a greater likelihood of finding those patients who can be identified as unable to survive resuscitation. Patients with severe extracranial hemorrhage and TBI who often have a combined trauma-induced coagulopathy caused by hypotension, hemorrhage, and coagulopathy of TBI frequently do not survive. These patients are much less likely to survive 72 h and should attract attention as an area of high potential to conserve blood products (2, 5, 6, 13, 14, 177). The presence of severe extracranial hemorrhage combined with TBI further allows for a more accurate prediction of futility using clinical, laboratory, and radiologic parameters for these SBTPs with TBI. Pathophysiologically, the patients with combined TBI and extracranial hemorrhage have dual causes for their coagulopathies, rendering them less likely to respond to heroic resuscitation efforts. These patients consume large volumes of blood products, and the number of blood products per hour serves as a surrogate for certain death when combined with other parameters, which provide a high level of certainty that these patients will die (2, 5, 6, 93, 177, 178). It should also be noted that there is a subset of patients with severe hiTBI who have such severe coagulopathy that they exhibit systemic bleeding that causes them to consume large quantities of blood products. The same criteria could be applied to this group of patients as well, even though they do not have extracranial injury (2, 5, 6, 78, 177).

**Figure 2:**
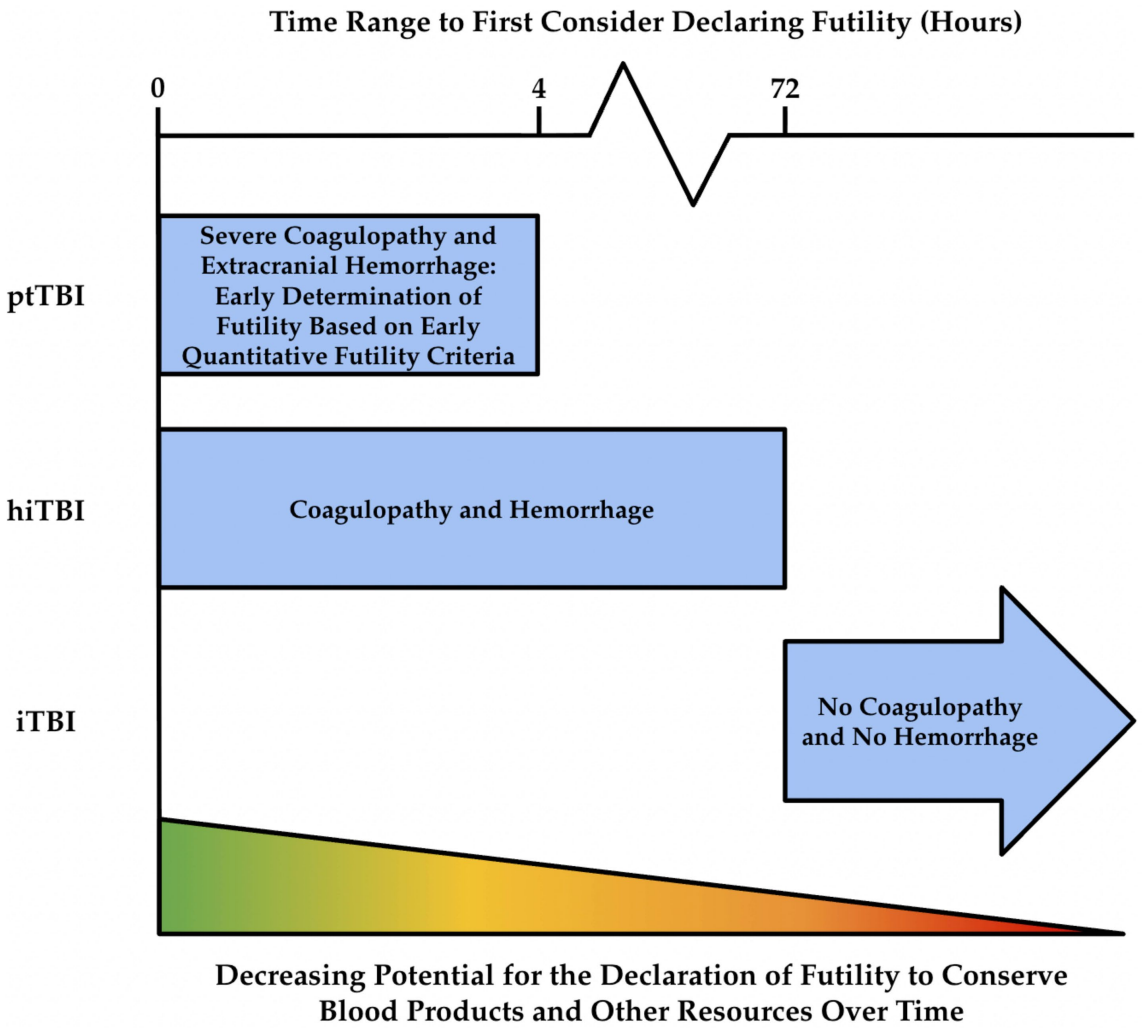
Time range after admission to first consider declaring futility (upper *x*-axis) for each type of TBI (*y*-axis). For patients with polytraumatic TBI (ptTBI) (or severe hemorrhagic isolated TBI (hiTBI) with profound hemorrhagic coagulopathy), futility can first be considered within 4 h because early predictors of futility, such as STOP, FCOPCA, and FoRM are based on 4-h time scales and/or arrival laboratory values (5, 7, 11, 85). For patients with isolated TBI (iTBI), it is recommended to wait at least 72 h before futility is declared, as iTBI patients have a better chance of survival and do not consume large quantities of blood products (13–15). The lower *x*-axis indicates the potential of guidelines with 100% PPV and specificity to conserve blood products and other limited resources by predicting futility. If futility can be declared early (within 4 h), more resources can be conserved compared to declaring futility late.

Since a large percentage of blood product usage for TBI patients with or without severe extracranial injury occurs in the first 4 h and the traditional waiting period for determining futility for iTBI is 72 h, Figure 2 displays a timeline whereby early predictions of futility can preserve large quantities of blood products (2, 5–7, 11, 13–15, 23, 25, 76, 85, 162, 179–181). To date, the highest level of evidence for declaring futility that is available in the literature is a combination of clinical and laboratory markers, such as STOP, FCOPCA, and FoRM.

The utilization of these quantitative futility assessments may allow refinement of the accuracy of determining futility during FTOs. It is proposed that in the first 4 h of treating ptTBI patients, the traumatologist may assess the likelihood of survival based on the number of blood products transfused combined with other severity parameters. Because there is controversy regarding the utility of blood product cut-points to predict FR, joining the STOP and FCOPCA criteria, which do not include blood product use, with FoRM, which includes blood product use as a marker, allows the traumatologists to more accurately predict futility in the earlier stages of trauma resuscitation (2, 5–7, 11, 23, 25, 76, 77, 85). It is important to note that despite this attempt to arrive at certainty with criteria that display 100% PPV and specificity, the physician’s clinical gestalt still underlies this attempt to quantitatively define futility, since it is up to the physician how to apply STOP, FCOPCA, and FoRM to ptTBI patients. More research will be required to advance beyond the quantitative futility criteria on the levels of evidence for WLST for SBTPs pyramid and establish universal guidelines.

In summary, the prediction of futility during FTOs is a simultaneously performed two-tier process whereby evaluation of patients with extracranial and cranial injury as candidates for futility during MT relies on the identification of sTBI, as well as an analysis of the markers for the depth and duration of hemorrhagic shock as predicted by quantitative futility criteria. Recently, FR protocols have been proposed to combine the use of markers of the severity of TBI with those that properly reflect the depth and duration of shock for patients with hemorrhagic extracranial loss of blood (2, 5–7, 11, 102, 168). These proposals form the basis for further research that can lead to the development of guidelines that can confidently predict futility in SBTPs with TBI.

## 6 Discussion

Considering recent blood shortages, the topic of bedside prediction of futility when administering large quantities of blood products to SBTPs has become an increasingly crucial area of ethical and medical research. However, universally accepted guidelines that can predict futility in SBTPs with TBI with 100% PPV and specificity do not currently exist (1–11). Some of the first bedside quantitative tools to predict futility have been proposed for children, adolescents, and adults, and they include various unique markers that are relevant to TBI patients, such as the units of blood products transfused per hour, severity of TBI, age, GCS, presence of midline shift, craniectomy, and coagulopathy (5, 7, 11, 85). The attempts to predict futility based on combinations of these reliable markers are efforts to create quantitative futility algorithms. For SBTPs, the first step would be to focus on the identification of sTBI and then to adhere to the parameters promulgated by STOP, FCOPCA, and FoRM. Given the scarcity of literature regarding reliable parameters that predict futility in this specific group of patients, only patients with ptTBI or patients with hiTBI and severe coagulopathy should be gauged as candidates for early futility until universally accepted guidelines with 100% PPV and specificity may be developed (2, 5–7, 11, 85).

From an ethical standpoint, transparency needs to be guaranteed when adhering to these predictors of certain death for patients who will not receive blood products due to futility. This transparency requires open communication between the blood bankers and other stakeholders, such as emergency physicians and members of an ethics committee, when these FR policies are instituted (6, 41, 182). Such shared decision-making, which is the result of collaboration between many medical and ethical specialties, will allow for the development of coherent, evidence-based criteria that will help to resolve conflicts between clinicians and patients or their surrogates and to positively redefine the process of declaring futility.

## Futile Indicators for Stopping Transfusion in Trauma (FISTT) Collaborative Group

The members of the FISTT Collaborative Group are Marie Karam, Jenny Chen, Samuel Thomas, Miguel Velasco, Afsheen Mansoori, Molly Feller, Mark Fox, Ernest Moore, Hunter Moore, Peter Moore, Vraj Patel, Jeffery Bao, Connor Schmitt, Joseph Robuck, Aleksey Zielinski, Daniel Lewandowski, Connor Bunch, Mark Walsh, Scott Thomas, Daniel Fulkerson, Joseph Miller, Mahmoud Al-Fadhl, Aadil Qasim, Reagan Ludwig, Aayan Siddiqi, Dan Waxman, Qamar Mohammad Ayoub, Hau Kwaan, Quincy Tran, Toby Brenner, Jack Langford, Alexander Mehreteab, Anne Grisoli, Kathleen Kelly, Nuha Zackariya, Mia Aboukhaled, Sarah George, Evfrosiniia Mozhaeva, Saniya Zackariya, Manaal Buchh, Haneefa Raza, Christopher Steinberg, Bilal Zackariya, Jonathan Zhao, Jason Mbianda, Leena Khan, Maleha Khan, Eshaal Kizilbash, Elaine Eder, Kevin Taubman, and Nicholas Kerr.

## Glossary

AIS: Abbreviated Injury Scale
APACHE II: Acute Physiology and Chronic Health Evaluation II
CAPSO: Commands, Age, Pulse rate, Systolic blood pressure, and peripheral Oxygen saturation
DCD: Donation after circulatory death
EBM: Evidence-based medicine
FCOPCA: Futility Cut-Off Points for Children and Adolescents
FFP: Fresh frozen plasma
FORECAST: Focused Outcomes Research in Emergency Care in Acute respiratory distress syndrome, Sepsis, and Trauma
FoRM: Futility of Resuscitation Measure
FR: Futile resuscitation
FTO: Futility time-out
GCS: Glasgow Coma Scale
hiTBI: Hemorrhagic isolated traumatic brain injury
INR: International normalized ratio
ISS: Injury Severity Score
iTBI: Isolated traumatic brain injury
LY30: Percent amplitude reduction of the clot 30 min after maximum amplitude
MT: Massive transfusion
PPV: Positive predictive value
PRBC: Packed red blood cell
PROPPR: Pragmatic, Randomized Optimal Platelet and Plasma Ratios
ptTBI: Polytraumatic traumatic brain injury
qEMAT: Quick Elderly Mortality After Trauma
ROSC: Return of spontaneous circulation
SBP: Systolic blood pressure
SBTP: Severely bleeding trauma patient
SOFA: Sequential Organ Failure Assessment
sTBI: Severe traumatic brain injury
STOP: Suspension of Transfusion and Other Procedures
TBI: Traumatic brain injury
TRIAGES: Trauma Rating Index in Age, Glasgow Coma Scale, Respiratory rate, and Systolic blood pressure score
TRISS: Trauma and Injury Severity Score
U: Units
WLST: Withdrawal of life-sustaining treatment

## References

[1] Williamson T Ryser MD Ubel PA Abdelgadir J Spears CA Liu B . Withdrawal of life-supporting treatment in severe traumatic brain injury. JAMA Surg. (2020) 155:723–31. doi: 10.1001/jamasurg.2020.1790, 32584926 PMC7301301

[2] Al-Fadhl MD Karam MN Chen J Zackariya SK Lain MC Bales JR . Traumatic brain injury as an independent predictor of futility in the early resuscitation of patients in hemorrhagic shock. J Clin Med. (2024) 13:3915. doi: 10.3390/jcm13133915, 38999481 PMC11242176

[3] Bosslet GT Pope TM Rubenfeld GD Lo B Truog RD Rushton CH . An official Ats/Aacn/Accp/Esicm/Sccm policy statement: responding to requests for potentially inappropriate treatments in intensive care units. Am J Respir Crit Care Med. (2015) 191:1318–30. doi: 10.1164/rccm.201505-0924ST, 25978438

[4] Nates JL Nunnally M Kleinpell R Blosser S Goldner J Birriel B . Icu admission, discharge, and triage guidelines: a framework to enhance clinical operations, development of institutional policies, and further research. Crit Care Med. (2016) 44:1553–602. doi: 10.1097/ccm.0000000000001856, 27428118

[5] Van Gent JM Clements TW Lubkin DT Wade CE Cardenas JC Kao LS . Predicting futility in severely injured patients: using arrival lab values and physiology to support evidence-based resource stewardship. J Am Coll Surg. (2023) 236:874–80. doi: 10.1097/XCS.0000000000000563, 36728085

[6] Walsh MM Fox MD Moore EE Johnson JL Bunch CM Miller JB . Markers of futile resuscitation in traumatic hemorrhage: a review of the evidence and a proposal for futility time-outs during massive transfusion. J Clin Med. (2024) 13:4684. doi: 10.3390/jcm13164684, 39200824 PMC11355875

[7] Bhogadi SK Ditillo M Khurshid MH Stewart C Hejazi O Spencer AL . Development and validation of futility of resuscitation measure in older adult trauma patients. J Surg Res. (2024) 301:591–8. doi: 10.1016/j.jss.2024.07.019, 39094517

[8] Turgeon AF Lauzier F Simard JF Scales DC Burns KE Moore L . Mortality associated with withdrawal of life-sustaining therapy for patients with severe traumatic brain injury: a Canadian multicentre cohort study. CMAJ. (2011) 183:1581–8. doi: 10.1503/cmaj.101786, 21876014 PMC3185074

[9] Haddam M Kubacsi L Hamada S Harrois A James A Langeron O . Withholding and withdrawal of life-sustaining therapy in 8569 trauma patients: a multicentre, analytical registry study. Eur J Anaesthesiol. (2022) 39:418–26. doi: 10.1097/EJA.0000000000001671 35166244

[10] Sullivan MD Owattanapanich N Schellenberg M Matsushima K Lewis MR Lam L . Examining the independent risk factors for withdrawal of life sustaining treatment in trauma patients. Injury. (2023) 54:111088. doi: 10.1016/j.injury.2023.111088, 37833232

[11] Cotton BA. Facing futility in hemorrhagic shock: when to say 'when' in children and adults. Trauma Surg Acute Care Open. (2024) 9:e001448. doi: 10.1136/tsaco-2024-001448, 38646027 PMC11029276

[12] Livingston DH Mosenthal AC. Withdrawing life-sustaining therapy for patients with severe traumatic brain injury. CMAJ. (2011) 183:1570–1. doi: 10.1503/cmaj.110974, 21876016 PMC3185069

[13] Miranda SP Morris RS Rabas M Creutzfeldt CJ Cooper Z. Early shared decision-making for older adults with traumatic brain injury: using time-limited trials and understanding their limitations. Neurocrit Care. (2023) 39:284–93. doi: 10.1007/s12028-023-01764-8, 37349599

[14] Souter MJ Blissitt PA Blosser S Bonomo J Greer D Jichici D . Recommendations for the critical Care Management of Devastating Brain Injury: prognostication, psychosocial, and ethical management: a position statement for healthcare professionals from the Neurocritical care society. Neurocrit Care. (2015) 23:4–13. doi: 10.1007/s12028-015-0137-6, 25894452

[15] American College of Surgeons Trauma Quality Improvement Program. Best Practices in the Management of Traumatic Brain Injury. Chicago: ACS Committee on Trauma (2015). p. 3–23.

[16] Tran A Saigle V Manhas N McIntyre L Turgeon AF Lauzier F . Association of age with death and withdrawal of life-sustaining therapy after severe traumatic brain injury. Can J Surg. (2023) 66:E348–55. doi: 10.1503/cjs.013721, 37402559 PMC10322160

[17] Eagle SR Nwachuku E Elmer J Deng H Okonkwo DO Pease M. Performance of crash and impact prognostic models for traumatic brain injury at 12 and 24 months post-injury. Neurotrauma Rep. (2023) 4:118–23. doi: 10.1089/neur.2022.0082, 36895818 PMC9989509

[18] Steyerberg EW Mushkudiani N Perel P Butcher I Lu J McHugh GS . Predicting outcome after traumatic brain injury: development and international validation of prognostic scores based on admission characteristics. PLoS Med. (2008) 5:e165. doi: 10.1371/journal.pmed.0050165, 18684008 PMC2494563

[19] MRC CRASH Trial Collaborators Perel P Arango M Clayton T Edwards P Komolafe E . Predicting outcome after traumatic brain injury: practical prognostic models based on large cohort of international patients. BMJ. (2008) 336:425–9. doi: 10.1136/bmj.39461.643438.25, 18270239 PMC2249681

[20] CRASH trial management group. The crash trial protocol (corticosteroid randomisation after significant head injury). BMC Emerg Med. (2001) 1:1–10. doi: 10.1186/1471-227X-1-1 11439175 PMC33506

[21] Wilkinson D. The self-fulfilling prophecy in intensive care. Theor Med Bioeth. (2009) 30:401–10. doi: 10.1007/s11017-009-9120-6, 19943193

[22] Ahmadi S Sarveazad A Babahajian A Ahmadzadeh K Yousefifard M. Comparison of Glasgow coma scale and full outline of unresponsiveness score for prediction of in-hospital mortality in traumatic brain injury patients: a systematic review and Meta-analysis. Eur J Trauma Emerg Surg. (2023) 49:1693–706. doi: 10.1007/s00068-022-02111-w, 36152069

[23] Loudon AM Rushing AP Hue JJ Ziemak A Sarode AL Moorman ML. When is enough enough? Odds of survival by unit transfused. J Trauma Acute Care Surg. (2023) 94:205–11. doi: 10.1097/TA.0000000000003835 36694331

[24] Clements T McCoy C Assen S Cardenas J Wade C Meyer D . The prehospital use of younger age whole blood is associated with an improved arrival coagulation profile. J Trauma Acute Care Surg. (2021) 90:607–14. doi: 10.1097/ta.0000000000003058, 33405468

[25] Clements TW Van Gent JM Lubkin DE Wandling MW Meyer DE Moore LJ . The reports of my death are greatly exaggerated: an evaluation of futility cut-points in massive transfusion. J Trauma Acute Care Surg. (2023) 95:685–90. doi: 10.1097/ta.0000000000003980 37125814

[26] Farrell MS Moore EE Thomas AV Coleman JR Thomas S Vande Lune S . Death diamond tracing on thromboelastography as a marker of poor survival after trauma. Am Surg. (2022) 88:1689–93. doi: 10.1177/0003134821998684 33629880

[27] Moore EE Moore HB Thomas SG Farrell MS Sixta S Coleman JR . Serial "death diamond" Tegs are a bedside Indicator of futile resuscitation during massive transfusion. J Trauma Acute Care Surg. (2023) 95:e19–21. doi: 10.1097/ta.0000000000003941, 37125795 PMC10476588

[28] Torres CM Kent A Scantling D Joseph B Haut ER Sakran JV. Association of Whole Blood with survival among patients presenting with severe hemorrhage in us and Canadian adult civilian trauma centers. JAMA Surg. (2023) 158:532–40. doi: 10.1001/jamasurg.2022.6978, 36652255 PMC9857728

[29] Torres CM Kenzik KM Saillant NN Scantling DR Sanchez SE Brahmbhatt TS . Timing to first whole blood transfusion and survival following severe hemorrhage in trauma patients. JAMA Surg. (2024) 159:374–81. doi: 10.1001/jamasurg.2023.7178, 38294820 PMC10831629

[30] Hashmi ZG Chehab M Nathens AB Joseph B Bank EA Jansen JO . Whole truths but half the blood: addressing the gap between the evidence and practice of pre-hospital and in-hospital blood product use for trauma resuscitation. Transfusion. (2021) 61:S348–53. doi: 10.1111/trf.16515 34086349

[31] Dhillon NK Kwon J Coimbra R. Fluid resuscitation in trauma: what you need to know. J Trauma Acute Care Surg. (2025) 98:20–9. doi: 10.1097/ta.0000000000004456, 39213260

[32] Holcomb JB Tilley BC Baraniuk S Fox EE Wade CE Podbielski JM . Transfusion of plasma, platelets, and red blood cells in a 1:1:1 vs a 1:1:2 ratio and mortality in patients with severe trauma: the Proppr randomized clinical trial. JAMA. (2015) 313:471–82. doi: 10.1001/jama.2015.12, 25647203 PMC4374744

[33] Mladinov D Frank SM. Massive transfusion and severe blood shortages: establishing and implementing predictors of futility. Br J Anaesth. (2022) 128:e71–4. doi: 10.1016/j.bja.2021.10.013, 34794769

[34] Saillant NN Kornblith LZ Moore H Barrett C Schreiber MA Cotton BA . The National Blood Shortage-an Impetus for change. Ann Surg. (2022) 275:641–3. doi: 10.1097/sla.0000000000005393, 35081570 PMC9055632

[35] Doughty H Green L Callum J Murphy MF. Triage tool for the rationing of blood for massively bleeding patients during a severe National Blood Shortage: guidance from the National Blood Transfusion Committee. Br J Haematol. (2020) 191:340–6. doi: 10.1111/bjh.16736, 32436251 PMC7280686

[36] Emanuel EJ Persad G Upshur R Thome B Parker M Glickman A . Fair allocation of scarce medical resources in the time of Covid-19. N Engl J Med. (2020) 382:2049–55. doi: 10.1056/NEJMsb2005114 32202722

[37] Kim JS Casem CF Baral E Inaba K Kuza CM. Narrative review: is there a transfusion cutoff value after which nonsurvivability is inevitable in trauma patients receiving ultramassive transfusion? Anesth Analg. (2023) 137:354–64. doi: 10.1213/ANE.0000000000006504 37115716

[38] Nakashima B Schellenberg M Gold AI Matsushima K Martin MJ Inaba K. Resuscitative thoracotomy for traumatic cardiac arrest: potential impact of resource constraint on outcomes and blood product utilization. J Surg Res. (2024) 295:683–9. doi: 10.1016/j.jss.2023.11.063, 38128347

[39] Ngo A Masel D Cahill C Blumberg N Refaai MA. Blood banking and transfusion medicine challenges during the COVID-19 pandemic. Clin Lab Med. (2020) 40:587–601. doi: 10.1016/j.cll.2020.08.013, 33121624 PMC7414314

[40] Riley W Love K McCullough J. Public policy impact of the Covid-19 pandemic on blood supply in the United States. Am J Public Health. (2021) 111:860–6. doi: 10.2105/ajph.2021.306157, 33734852 PMC8034029

[41] Lo BD Merkel KR Dougherty JL Kajstura TJ Cruz NC Sikorski RA . Assessing predictors of futility in patients receiving massive transfusions. Transfusion. (2021) 61:2082–9. doi: 10.1111/trf.16410, 33955577

[42] Stopenski S Grigorian A Inaba K Lekawa M Matsushima K Schellenberg M . Prehospital variables alone can predict mortality after blunt trauma: a novel scoring tool. Am Surg. (2021) 87:1638–43. doi: 10.1177/00031348211024192, 34128401

[43] Quintana MT Zebley JA Vincent A Chang P Estroff J Sarani B . Cresting mortality: defining a plateau in ongoing massive transfusion. J Trauma Acute Care Surg. (2022) 93:43–51. doi: 10.1097/ta.0000000000003641, 35393379

[44] Goettler CE Waibel BH Goodwin J Watkins F Toschlog EA Sagraves SG . Trauma intensive care unit survival: how good is an educated guess? J Trauma. (2010) 68:1279–87. doi: 10.1097/TA.0b013e3181de3b99 20539170

[45] Wongchareon K Thompson HJ Mitchell PH Barber J Temkin N. Impact and crash prognostic models for traumatic brain injury: external validation in a south-American cohort. Inj Prev. (2020) 26:546–54. doi: 10.1136/injuryprev-2019-043466, 31959626

[46] Karabacak M Jagtiani P Dams-O'Connor K Legome E Hickman ZL Margetis K. The most (mortality score for Tbi): a novel prediction model beyond crash-basic and impact-core for isolated traumatic brain injury. Injury. (2025) 56:111956. doi: 10.1016/j.injury.2024.111956, 39428266

[47] Joseph B Saljuqi AT Amos JD Teichman A Whitmill ML Anand T . Prospective validation and application of the trauma-specific frailty index: results of an American Association for the Surgery of Trauma multi-institutional observational trial. J Trauma Acute Care Surg. (2023) 94:36–44. doi: 10.1097/ta.0000000000003817, 36279368

[48] Joseph B Pandit V Zangbar B Kulvatunyou N Tang A O'Keeffe T . Validating trauma-specific frailty index for geriatric trauma patients: a prospective analysis. J Am Coll Surg. (2014) 219:10–7e1. doi: 10.1016/j.jamcollsurg.2014.03.020 24952434

[49] Shiber J Fontane E Patel J Akinleye A Kerwin A Chiu W . Gestalt clinical severity score (Gcss) as a predictor of patient severity of illness or injury. Am J Emerg Med. (2023) 66:11–5. doi: 10.1016/j.ajem.2023.01.005, 36640694

[50] Vorbeck J Bachmann M Düsing H Hartensuer R. Mortality risk factors of severely injured Polytrauma patients (prehospital mortality prediction score). J Clin Med. (2023) 12:4724. doi: 10.3390/jcm12144724, 37510839 PMC10380896

[51] Egglestone R Sparkes D Dushianthan A. Prediction of mortality in critically-ill elderly trauma patients: a single Centre retrospective observational study and comparison of the performance of trauma scores. Scand J Trauma Resusc Emerg Med. (2020) 28:95. doi: 10.1186/s13049-020-00788-9, 32967736 PMC7510154

[52] Palmer CS Gabbe BJ Cameron PA. Defining major trauma using the 2008 abbreviated injury scale. Injury. (2016) 47:109–15. doi: 10.1016/j.injury.2015.07.003, 26283084

[53] Kunitake RC Kornblith LZ Cohen MJ Callcut RA. Trauma early mortality prediction tool (tempt) for assessing 28-day mortality. Trauma Surg Acute Care Open. (2018) 3:e000131. doi: 10.1136/tsaco-2017-000131, 29766125 PMC5887834

[54] Shiraishi A Otomo Y Yoshikawa S Morishita K Roberts I Matsui H. Derivation and validation of an easy-to-compute trauma score that improves prognostication of mortality or the trauma rating index in age, Glasgow coma scale, respiratory rate and systolic blood pressure (Triages) score. Crit Care. (2019) 23:365. doi: 10.1186/s13054-019-2636-x, 31752938 PMC6868841

[55] Sefrioui I Amadini R Mauro J El Fallahi A Gabbrielli M. Survival prediction of trauma patients: a study on us National Trauma Data Bank. Eur J Trauma Emerg Surg. (2017) 43:805–22. doi: 10.1007/s00068-016-0757-3, 28229175

[56] Hörauf JA Woschek M Schindler CR Verboket RD Lustenberger T Marzi I . Settlement is at the end-common trauma scores require a critical reassessment due to the possible dynamics of traumatic brain injuries in patients' clinical course. J Clin Med. (2024) 13:3333. doi: 10.3390/jcm13113333, 38893044 PMC11173217

[57] Millin MG Galvagno SM Khandker SR Malki A Bulger EM. Withholding and termination of resuscitation of adult cardiopulmonary arrest secondary to trauma: resource document to the joint Naemsp-Acscot position statements. J Trauma Acute Care Surg. (2013) 75:459–67. doi: 10.1097/TA.0b013e31829cfaea, 24089117

[58] National Association of EMS Physicians and American College of Surgeons Committee on Trauma. Withholding of resuscitation for adult traumatic cardiopulmonary arrest. Prehosp Emerg Care. (2013) 17:291. doi: 10.3109/10903127.2012.755586 23327549

[59] Kersjes E Smith LB. How should decision science inform scarce blood product allocation? AMA J Ethics. (2019) 21:E852–7. doi: 10.1001/amajethics.2019.852, 31651384

[60] Ardagh M. Futility has no utility in resuscitation medicine. J Med Ethics. (2000) 26:396–9. doi: 10.1136/jme.26.5.396, 11055046 PMC1733283

[61] Bosslet GT Kesecioglu J White DB. How should clinicians respond to requests for potentially inappropriate treatment? Intensive Care Med. (2016) 42:422–5. doi: 10.1007/s00134-015-4192-4, 26762106

[62] Cantor MD Braddock CH 3rd Derse AR Edwards DM Logue GL Nelson W . Do-not-resuscitate orders and medical futility. Arch Intern Med. (2003) 163:2689–94. doi: 10.1001/archinte.163.22.2689 14662622

[63] Nirula R Gentilello LM. Futility of resuscitation criteria for the "young" old and the "old" old trauma patient: a National Trauma Data Bank analysis. J Trauma. (2004) 57:37–41. doi: 10.1097/01.ta.0000128236.45043.6a 15284545

[64] Duvall DB Zhu X Elliott AC Wolf SE Rhodes RL Paulk ME . Injury severity and comorbidities alone do not predict futility of care after geriatric trauma. J Palliat Med. (2015) 18:246–50. doi: 10.1089/jpm.2014.0336, 25494453 PMC4347887

[65] Yekefallah L Ashktorab T Manoochehri H Majd HA. Developing a tool for evaluation of causes of futile Care in Intensive Care Units. Iran J Nurs Midwifery Res. (2019) 24:56–60. doi: 10.4103/ijnmr.IJNMR_146_17, 30622579 PMC6298164

[66] White B Willmott L Close E Shepherd N Gallois C Parker MH . What does "futility" mean? An empirical study of doctors' perceptions. Med J Aust. (2016) 204:318. doi: 10.5694/mja15.01103, 27125807

[67] Cook C. Is clinical gestalt good enough? J Man Manip Ther. (2009) 17:6–7. doi: 10.1179/106698109790818223, 20046560 PMC2704346

[68] Kabrhel C Camargo CA Jr Goldhaber SZ. Clinical gestalt and the diagnosis of pulmonary embolism: does experience matter? Chest. (2005) 127:1627–30. doi: 10.1378/chest.127.5.1627, 15888838

[69] Kempainen RR Migeon MB Wolf FM. Understanding our mistakes: a primer on errors in clinical reasoning. Med Teach. (2003) 25:177–81. doi: 10.1080/0142159031000092580, 12745527

[70] Baik D Yeom SR Park SW Cho Y Yang WT Kwon H . The addition of Rotem parameter did not significantly improve the massive transfusion prediction in severe trauma patients. Emerg Med Int. (2022) 2022:7219812. doi: 10.1155/2022/7219812, 36285178 PMC9588372

[71] Nunez TC Voskresensky IV Dossett LA Shinall R Dutton WD Cotton BA. Early prediction of massive transfusion in trauma: simple as Abc (assessment of blood consumption)? J Trauma. (2009) 66:346–52. doi: 10.1097/TA.0b013e3181961c35, 19204506

[72] Pommerening MJ Goodman MD Holcomb JB Wade CE Fox EE Del Junco DJ . Clinical gestalt and the prediction of massive transfusion after trauma. Injury. (2015) 46:807–13. doi: 10.1016/j.injury.2014.12.026, 25682314 PMC4800814

[73] White DB Pope TM. "Medical futility and potentially inappropriate treatment". In: Youngner SJ Arnold RM, editors. The Oxford Handbook of Ethics at the End of Life. Oxford: Oxford University Press (2016). p. 65–86.

[74] Malhotra AK Shakil H Essa A Mathieu F Taran S Badhiwala J . Influence of health insurance on withdrawal of life sustaining treatment for patients with isolated traumatic brain injury: a retrospective multi-center observational cohort study. Crit Care. (2024) 28:251. doi: 10.1186/s13054-024-05027-6, 39026325 PMC11264615

[75] van Veen E van der Jagt M Citerio G Stocchetti N Gommers D Burdorf A . Occurrence and timing of withdrawal of life-sustaining measures in traumatic brain injury patients: a center-Tbi study. Intensive Care Med. (2021) 47:1115–29. doi: 10.1007/s00134-021-06484-1, 34351445 PMC8486724

[76] Van Gent JM Clements TW Rosario-Rivera BL Wisniewski SR Cannon JW Schreiber MA . The inability to predict futility in hemorrhaging trauma patients using 4-hour transfusion volumes and rates. J Trauma Acute Care Surg. (2025) 98:39760660:236–42. doi: 10.1097/ta.0000000000004541 39760660

[77] Major FR Pickering TA Stefanescu K Singh M Clark DH Inaba K . A retrospective study of Ultramassive transfusion in trauma patients: is there a value after which additional transfusions are futile? Anesth Analg. (2025) 141:1126–36. doi: 10.1213/ane.0000000000007569, 40445862

[78] Wada T Shiraishi A Gando S Yamakawa K Fujishima S Saitoh D . Pathophysiology of coagulopathy induced by traumatic brain injury is identical to that of disseminated intravascular coagulation with Hyperfibrinolysis. Front Med (Lausanne). (2021) 8:767637. doi: 10.3389/fmed.2021.767637, 34869481 PMC8634586

[79] Morris RS Milia D Glover J Napolitano LM Chen B Lindemann E . Predictors of elderly mortality after trauma: a novel outcome score. J Trauma Acute Care Surg. (2020) 88:416–24. doi: 10.1097/ta.0000000000002569, 31895331

[80] Hosseinpour H Anand T Bhogadi SK Colosimo C El-Qawaqzeh K Spencer AL . Emergency department shock index outperforms prehospital and delta shock indices in predicting outcomes of trauma patients. J Surg Res. (2023) 291:204–12. doi: 10.1016/j.jss.2023.05.008, 37451172

[81] Li Y Wang L Liu Y Zhao Y Fan Y Yang M . Development and validation of a simplified prehospital triage model using neural network to predict mortality in trauma patients: the ability to follow commands, age, pulse rate, systolic blood pressure and peripheral oxygen saturation (Capso) model. Front Med (Lausanne). (2021) 8:810195. doi: 10.3389/fmed.2021.810195, 34957169 PMC8709125

[82] Choi Y Park JH Hong KJ Ro YS Song KJ Shin SD. Development and validation of a prehospital-stage prediction tool for traumatic brain injury: a multicentre retrospective cohort study in Korea. BMJ Open. (2022) 12:e055918. doi: 10.1136/bmjopen-2021-055918, 35022177 PMC8756263

[83] Jiang D Chen T Yuan X Shen Y Huang Z. Predictive value of the trauma rating index in age, Glasgow coma scale, respiratory rate and systolic blood pressure score (Triages) and revised trauma score (Rts) for the short-term mortality of patients with isolated traumatic brain injury. Am J Emerg Med. (2023) 71:175–81. doi: 10.1016/j.ajem.2023.06.030, 37421814

[84] Benhamed A Emond M Mercier E Heidet M Gauss T Saint-Supery P . Accuracy of a prehospital triage protocol in predicting in-hospital mortality and severe trauma cases among older adults. Int J Environ Res Public Health. (2023) 20:1975. doi: 10.3390/ijerph20031975, 36767343 PMC9916137

[85] Kalkwarf KJ Jensen SD Allukian M III Harting T Cox CS Fox EE . Can we identify futility in kids? An evaluation of admission parameters predicting 100% mortality in 1,292 severely injured children. J Am Coll Surg. (2018) 226:662–7. doi: 10.1016/j.jamcollsurg.2017.12.034, 29325878

[86] Dzik W. Misunderstanding the Proppr trial. Transfusion. (2017) 57:2056. doi: 10.1111/trf.14200, 28782822

[87] Holcomb JB Hess JR Group PS. Response to:“misunderstanding the Proppr trial”. Transfusion. (2017) 57:2057–8. doi: 10.1111/trf.14197 28782821

[88] Walsh M Fries D Moore E Moore H Thomas S Kwaan HC . "Whole blood for civilian urban trauma resuscitation: historical, present, and future considerations". In: Seminars in Thrombosis and Hemostasis. New York City: Thieme Medical Publishers (2020) 10.1055/s-0040-1702174 32160645

[89] Walsh M Moore EE Moore HB Thomas S Kwaan HC Speybroeck J . Whole blood, fixed ratio, or goal-directed blood component therapy for the initial resuscitation of severely hemorrhaging trauma patients: a narrative review. J Clin Med. (2021) 10:320. doi: 10.3390/jcm10020320, 33477257 PMC7830337

[90] Malhotra AK Shakil H Smith CW Sader N Ladha K Wijeysundera DN . Withdrawal of life-sustaining treatment for pediatric patients with severe traumatic brain injury. JAMA Surg. (2024) 159:287–96. doi: 10.1001/jamasurg.2023.6531, 38117514 PMC10733846

[91] Capizzani AR Drongowski R Ehrlich PF. Assessment of termination of trauma resuscitation guidelines: are children small adults? J Pediatr Surg. (2010) 45:903–7. doi: 10.1016/j.jpedsurg.2010.02.014, 20438923

[92] Reppucci ML Pickett K Stevens J Phillips R Recicar J Annen K . Massive transfusion in pediatric trauma-does more blood predict mortality? J Pediatr Surg. (2022) 57:308–13. doi: 10.1016/j.jpedsurg.2021.09.051, 34736771

[93] Maegele M Schöchl H Menovsky T Maréchal H Marklund N Buki A . Coagulopathy and haemorrhagic progression in traumatic brain injury: advances in mechanisms, diagnosis, and management. Lancet Neurol. (2017) 16:630–47. doi: 10.1016/s1474-4422(17)30197-7, 28721927

[94] Cook AC Joseph B Inaba K Nakonezny PA Bruns BR Kerby JD . Multicenter external validation of the geriatric trauma outcome score: a study by the prognostic assessment of life and limitations after trauma in the elderly (palliate) consortium. J Trauma Acute Care Surg. (2016) 80:204–9. doi: 10.1097/ta.0000000000000926, 26595708

[95] Park J Lee Y. Predicting mortality of Korean geriatric trauma patients: a comparison between geriatric trauma outcome score and trauma and injury severity score. Yonsei Med J. (2022) 63:88–94. doi: 10.3349/ymj.2022.63.1.88, 34913288 PMC8688368

[96] Ravindranath S Ho KM Rao S Nasim S Burrell M. Validation of the geriatric trauma outcome scores in predicting outcomes of elderly trauma patients. Injury. (2021) 52:154–9. doi: 10.1016/j.injury.2020.09.056, 33082025

[97] Zhuang Y Feng Q Tang H Wang Y Li Z Bai X. Predictive value of the geriatric trauma outcome score in older patients after trauma: a retrospective cohort study. Int J Gen Med. (2022) 15:4379–90. doi: 10.2147/IJGM.S362752, 35493196 PMC9045832

[98] Scherer J Kalbas Y Ziegenhain F Neuhaus V Lefering R Teuben M . The Gertality score: the development of a simple tool to help predict in-hospital mortality in geriatric trauma patients. J Clin Med. (2021) 10:1362. doi: 10.3390/jcm10071362, 33806240 PMC8037079

[99] Zhao FZ Wolf SE Nakonezny PA Minhajuddin A Rhodes RL Paulk ME . Estimating geriatric mortality after injury using age, injury severity, and performance of a transfusion: the geriatric trauma outcome score. J Palliat Med. (2015) 18:677–81. doi: 10.1089/jpm.2015.0027, 25974408 PMC4522950

[100] Chow J Kuza CM. Predicting mortality in elderly trauma patients: a review of the current literature. Curr Opin Anaesthesiol. (2022) 35:160–5. doi: 10.1097/ACO.0000000000001092, 35025820

[101] El-Qawaqzeh K Magnotti LJ Hosseinpour H Nelson A Spencer AL Anand T . Geriatric trauma, frailty, and Acs trauma center verification level: are there any correlations with outcomes? Injury. (2024) 55:110972. doi: 10.1016/j.injury.2023.110972, 37573210

[102] Jeon S Lee GJ Lee M Choi KK Lee SH Cho J . Predictive limitations of the geriatric trauma outcome score: a retrospective analysis of mortality in elderly patients with multiple traumas and severe traumatic brain injury. Diagnostics. (2025) 15:586. doi: 10.3390/diagnostics15050586, 40075833 PMC11899710

[103] Barbosa RR Rowell SE Diggs BS Schreiber MA Group TO. Profoundly abnormal initial physiologic and biochemical data cannot be used to determine futility in massively transfused trauma patients. J Trauma Injury Infect Crit Care. (2011) 71:S364–9. doi: 10.1097/TA.0b013e318227f170, 21814105

[104] Dorken Gallastegi A Secor JD Maurer LR Dzik WS Saillant NN Hwabejire JO . Role of transfusion volume and transfusion rate as markers of futility during Ultramassive blood transfusion in trauma. J Am Coll Surg. (2022) 235:468–80. doi: 10.1097/xcs.0000000000000268, 35972167

[105] Hamidi M Zeeshan M Kulvatunyou N Adun E O'Keeffe T Zakaria ER . Outcomes after massive transfusion in trauma patients: variability among trauma centers. J Surg Res. (2019) 234:110–5. doi: 10.1016/j.jss.2018.09.018, 30527461

[106] Huber-Wagner S Qvick M Mussack T Euler E Kay MV Mutschler W . Massive blood transfusion and outcome in 1062 Polytrauma patients: a prospective study based on the trauma registry of the German trauma society. Vox Sang. (2007) 92:69–78. doi: 10.1111/j.1423-0410.2006.00858.x, 17181593

[107] Mitra B Mori A Cameron PA Fitzgerald M Paul E Street A. Fresh frozen plasma (Ffp) use during massive blood transfusion in trauma resuscitation. Injury. (2010) 41:35–9. doi: 10.1016/j.injury.2009.09.029, 19833331

[108] Mitra B Olaussen A Cameron PA O'Donohoe T Fitzgerald M. Massive blood transfusions post trauma in the elderly compared to younger patients. Injury. (2014) 45:1296–300. doi: 10.1016/j.injury.2014.01.016, 24560872

[109] Moore FA Nelson T McKinley BA Moore EE Nathens AB Rhee P . Massive transfusion in trauma patients: tissue hemoglobin oxygen saturation predicts poor outcome. J Trauma. (2008) 64:1010–23. doi: 10.1097/TA.0b013e31816a2417, 18404069

[110] Morris MC Niziolek GM Baker JE Huebner BR Hanseman D Makley AT . Death by decade: establishing a transfusion ceiling for futility in massive transfusion. J Surg Res. (2020) 252:139–46. doi: 10.1016/j.jss.2020.03.004, 32278968

[111] Mostafa G Gunter OL Norton HJ McElhiney BM Bailey DF Jacobs DG. Age, blood transfusion, and survival after trauma. Am Surg. (2004) 70:357–63. doi: 10.1177/000313480407000418, 15098792

[112] Sharpe JP Weinberg JA Magnotti LJ Croce MA Fabian TC. Toward a better definition of massive transfusion: focus on the interval of hemorrhage control. J Trauma Acute Care Surg. (2012) 73:1553–7. doi: 10.1097/TA.0b013e3182660119, 23032813

[113] Velmahos GC Chan L Chan M Tatevossian R Cornwell EE III Asensio JA . Is there a limit to massive blood transfusion after severe trauma? Arch Surg. (1998) 133:947–52. 9749845 10.1001/archsurg.133.9.947

[114] L'Huillier JC Hua S Logghe HJ Yu J Myneni AA Noyes K . Transfusion futility thresholds and mortality in geriatric trauma: does frailty matter? Am J Surg. (2024) 228:113–21. doi: 10.1016/j.amjsurg.2023.08.020, 37684168

[115] El-Qawaqzeh K Anand T Alizai Q Colosimo C Hosseinpour H Spencer A . Trauma in the geriatric and the super-geriatric: should they be treated the same? J Surg Res. (2024) 293:316–26. doi: 10.1016/j.jss.2023.09.015, 37806217

[116] Arslan A Flax L Fraser R Kanter M Simon R Caputo ND. Twenty-four-hour packed red blood cell requirement is the strongest independent prognostic marker of mortality in Ed trauma patients. Am J Emerg Med. (2016) 34:1121–4. doi: 10.1016/j.ajem.2016.03.036, 27066932

[117] Hertelendy AJ Ciottone GR Mitchell CL Gutberg J Burkle FM. Crisis standards of Care in a Pandemic: navigating the ethical, clinical, psychological and policy-making maelstrom. Int J Qual Health Care. (2021) 33:mzaa094. doi: 10.1093/intqhc/mzaa094, 33128564 PMC7454656

[118] Koeckerling D Pan D Mudalige NL Oyefeso O Barker J. Blood transfusion strategies and Ecmo during the Covid-19 pandemic. Lancet Respir Med. (2020) 8:e40. doi: 10.1016/S2213-2600(20)30173-9, 32305078 PMC7162621

[119] Ramanathan K Antognini D Combes A Paden M Zakhary B Ogino M . Planning and provision of Ecmo Services for Severe Ards during the Covid-19 pandemic and other outbreaks of emerging infectious diseases. Lancet Respir Med. (2020) 8:518–26. doi: 10.1016/S2213-2600(20)30121-1, 32203711 PMC7102637

[120] Stanworth SJ New HV Apelseth TO Brunskill S Cardigan R Doree C . Effects of the Covid-19 pandemic on supply and use of blood for transfusion. Lancet Haematol. (2020) 7:e756–64. doi: 10.1016/S2352-3026(20)30186-1, 32628911 PMC7333996

[121] Rawlings A Brandt L Ferreres A Asbun H Shadduck P. Ethical considerations for allocation of scarce resources and alterations in surgical care during a pandemic. Surg Endosc. (2021) 35:2217–22. doi: 10.1007/s00464-020-07629-x, 32399942 PMC7216853

[122] DeSimone RA Costa VA Kane K Sepulveda JL Ellsworth GB Gulick RM . Blood component utilization in Covid-19 patients in New York City: transfusions do not follow the curve. Transfusion. (2021) 61:692–8. doi: 10.1111/trf.16202, 33215718 PMC7753518

[123] Oh YJ Kim JY Suh JW Jeong Y Choi Y Lim HJ . Blood transfusion utilization in patients with severe coronavirus disease 2019 in the Republic of Korea: a Nationwide population-based study. J Clin Med. (2024) 13:7327. doi: 10.3390/jcm13237327, 39685786 PMC11642700

[124] Beauchamp T Childress J. Principles of biomedical ethics: marking its fortieth anniversary. Am J Bioeth. (2019) 19:9–12. doi: 10.1080/15265161.2019.1665402, 31647760

[125] Varkey B. Principles of clinical ethics and their application to practice. Med Princ Pract. (2021) 30:17–28. doi: 10.1159/000509119, 32498071 PMC7923912

[126] Zakrison TL Essig R Polcari A McKinley W Arnold D Beyene R . Review paper on penetrating brain injury: ethical quandaries in the Trauma Bay and beyond. Ann Surg. (2023) 277:66–72. doi: 10.1097/sla.0000000000005608, 35997268 PMC9762724

[127] Chin TL Moore EE Coors ME Chandler JG Ghasabyan A Harr JN . Exploring ethical conflicts in emergency trauma research: the combat (control of Major bleeding after trauma) study experience. Surgery. (2015) 157:10–9. doi: 10.1016/j.surg.2014.05.021, 25444222 PMC4261038

[128] Gillon R. Defending the four principles approach as a good basis for good medical practice and therefore for good medical ethics. J Med Ethics. (2015) 41:111–6. doi: 10.1136/medethics-2014-102282 25516950

[129] Aacharya RP Gastmans C Denier Y. Emergency department triage: an ethical analysis. BMC Emerg Med. (2011) 11:16. doi: 10.1186/1471-227x-11-16, 21982119 PMC3199257

[130] Smith LB Cooling L Davenport R. How do I allocate blood products at the end of life? An ethical analysis with suggested guidelines. Transfusion. (2013) 53:696–700. doi: 10.1111/j.1537-2995.2012.03658.x, 22519756

[131] Simpson O. Consent and assessment of capacity to decide or refuse treatment. Br J Nurs. (2011) 20:510–3. doi: 10.12968/bjon.2011.20.8.510, 21537284

[132] Carrese JA. Refusal of care: patients' well-being and physicians' ethical obligations: "but doctor, I want to go home". JAMA. (2006) 296:691–5. doi: 10.1001/jama.296.6.691, 16896112

[133] Truog RD. Is it always wrong to perform futile Cpr? N Engl J Med. (2010) 362:477–9. doi: 10.1056/NEJMp0908464, 20147712

[134] Tomlinson T Brody H. Futility and the ethics of resuscitation. JAMA. (1990) 264:1276–80. 2388379

[135] Vivas L Carpenter T. Meaningful futility: requests for resuscitation against medical recommendation. J Med Ethics. (2021) 47:654–6. doi: 10.1136/medethics-2020-106232, 32332150 PMC8479753

[136] Bester JC. The best interest standard is the best we have: why the harm principle and constrained parental autonomy cannot replace the best interest standard in pediatric ethics. J Clin Ethics. (2019) 30:223–31. doi: 10.1086/JCE2019303223, 31573966

[137] Tonelli MR Misak CJ. Compromised autonomy and the seriously ill patient. Chest. (2010) 137:926–31. doi: 10.1378/chest.09-1574, 20371527

[138] Suah A Angelos P. How should trauma patients' informed consent or refusal be regarded in a Trauma Bay or other emergency settings? AMA J Ethics. (2018) 20:425–30. doi: 10.1001/journalofethics.2018.20.5.ecas1-1805, 29763388

[139] Badrinathan A Ho VP Tinkoff G Houck O Vazquez D Gerrek M . Are we waiting for the sky to fall? Predictors of withdrawal of life-sustaining support in older trauma patients: a retrospective analysis. J Trauma Acute Care Surg. (2023) 94:385–91. doi: 10.1097/TA.0000000000003844, 36449699 PMC9974547

[140] Mosenthal AC. Dying of traumatic brain injury-palliative care too soon, or too late? JAMA Surg. (2020) 155:731. doi: 10.1001/jamasurg.2020.1810 32584939

[141] Kopar PK Visani A Squirrell K Brown DE. Addressing futility: a practical approach. Crit Care Explor. (2022) 4:e0706. doi: 10.1097/cce.0000000000000706, 35815180 PMC9257305

[142] Fritz Z Cox CL. Integrating philosophy, policy and practice to create a just and fair health service. J Med Ethics. (2020) 46:797–802. doi: 10.1136/medethics-2020-106853, 33028624 PMC7719902

[143] Malaiyandi DP Henderson GV Rubin MA. Transfusion of blood products in the Neurocritical care unit: an exploration of rationing and futility. Neurocrit Care. (2018) 28:296–301. doi: 10.1007/s12028-017-0478-4, 29288291

[144] Caplan A. Bioethics of organ transplantation. Cold Spring Harb Perspect Med. (2014) 4:a015685. doi: 10.1101/cshperspect.a015685, 24478386 PMC3935394

[145] Shafran D Kodish E Tzakis A. Organ shortage: the greatest challenge facing transplant medicine. World J Surg. (2014) 38:1650–7. doi: 10.1007/s00268-014-2639-3 24831673

[146] Neuberger J. Rationing life-saving resources--how should allocation policies be assessed in solid organ transplantation. Transpl Int. (2012) 25:3–6. doi: 10.1111/j.1432-2277.2011.01327.x, 21902728

[147] Tanaka T Lai JC Axelrod D Sewell D. Meld 3.I: a Bayesian updating to the model for end-stage liver disease. JHEP Rep. (2025) 7:101525. doi: 10.1016/j.jhepr.2025.101525 PMC12506478 41070101

[148] Kanwal F Hernaez R Liu Y Taylor TJ Rana A Kramer JR . Factors associated with access to and receipt of liver transplantation in veterans with end-stage liver disease. JAMA Intern Med. (2021) 181:949–59. doi: 10.1001/jamainternmed.2021.2051, 34028505 PMC8145153

[149] Kim CH Lee FG Beaman LR Meyer LE Diwan TS. Decision-making in transplant candidate selection committees: frameworks and other considerations. Mayo Clin Proc. (2025) 100:1402–10. doi: 10.1016/j.mayocp.2025.04.022, 40616583

[150] Park H Jung ES Oh JS Lee YM Lee JM. Organ donation after controlled circulatory death (Maastricht classification iii) following the withdrawal of life-sustaining treatment in Korea: a suggested guideline. Korean J Transplant. (2021) 35:71–6. doi: 10.4285/kjt.21.0004, 35769520 PMC9235338

[151] Lacroix JD Mahoney JE Knoll GA. Renal transplantation using non-heart-beating donors: a potential solution to the organ donor shortage in Canada. Can J Surg. (2004) 47:10–4. 14997918 PMC3211806

[152] Thuong M Ruiz A Evrard P Kuiper M Boffa C Akhtar MZ . New classification of donation after circulatory death donors definitions and terminology. Transpl Int. (2016) 29:749–59. doi: 10.1111/tri.12776, 26991858

[153] Morrison LJ Sandroni C Grunau B Parr M Macneil F Perkins GD . Organ donation after out-of-hospital cardiac arrest: a scientific statement from the international liaison committee on resuscitation. Circulation. (2023) 148:e120–46. doi: 10.1161/CIR.0000000000001125, 37551611

[154] D'Aragon F Lachance O Lafleur V Ortega-Deballon I Masse MH Trepanier G . Program of uncontrolled donation after circulatory death as potential solution to the shortage of organs: a Canadian single-center retrospective cohort study. Open Access Emerg Med. (2022) 14:413–20. doi: 10.2147/OAEM.S361930, 35958629 PMC9362902

[155] Simon JR Schears RM Padela AI. Donation after cardiac death and the emergency department: ethical issues. Acad Emerg Med. (2014) 21:79–86. doi: 10.1111/acem.12284, 24552527

[156] Tzeng WJ Tseng HY Hou TY Chou SE Su WT Hsu SY . From death triad to death tetrad-the addition of a hypotension component to the death triad improves mortality risk stratification in trauma patients: a retrospective cohort study. Diagnostics. (2022) 12:2885. doi: 10.3390/diagnostics12112885, 36428944 PMC9689469

[157] Agrawal S Magoon R Choudhary N Suresh V Kumar A Nagpal VK . Evidence-based medicine: a narrative review on the evolving opportunities and challenges. J Cardiac Crit Care TSS. (2023) 8:122–8. doi: 10.25259/jccc_51_2023

[158] Gelardi F Kirienko M Sollini M. Climbing the steps of the evidence-based medicine pyramid: highlights from annals of nuclear medicine 2019. Eur J Nucl Med Mol Imaging. (2021) 48:1293–301. doi: 10.1007/s00259-020-05073-6, 33150459

[159] Vatkar A Kale S Shyam A Srivastava S. Understanding the levels of evidence in medical research. J Orthopaedic Case Rep. (2025) 15:6–9. doi: 10.13107/jocr.2025.v15.i05.5534, 40351623 PMC12064251

[160] Collins J. Evidence-based medicine. J Am Coll Radiol. (2007) 4:551–4. doi: 10.1016/j.jacr.2006.12.007 17660119

[161] Bell C Prokopchuk-Gauk O Cload B Stirling A Davis PJ. Optimum accuracy of massive transfusion protocol activation: the clinician's view. Cureus. (2018) 10:e3688. doi: 10.7759/cureus.3688, 30761240 PMC6368427

[162] Boye M Py N Libert N Chrisment A Pissot M Dedome E . Step by step transfusion timeline and its challenges in trauma: a retrospective study in a level one trauma center. Transfusion. (2022) 62:S30–s42. doi: 10.1111/trf.16953, 35781713

[163] Bohm JK Guting H Thorn S Schafer N Rambach V Schochl H . Global characterisation of coagulopathy in isolated traumatic brain injury (ITBI): a center-TBI analysis. Neurocrit Care. (2021) 35:184–96. doi: 10.1007/s12028-020-01151-7, 33306177 PMC8285342

[164] Stolla M Zhang F Meyer MR Zhang J Dong JF. Current state of transfusion in traumatic brain injury and associated coagulopathy. Transfusion. (2019) 59:1522–8. doi: 10.1111/trf.15169, 30980753

[165] Samuels JM Moore EE Silliman CC Banerjee A Cohen MJ Ghasabyan A . Severe traumatic brain injury is associated with a unique coagulopathy phenotype. J Trauma Acute Care Surg. (2019) 86:686–93. doi: 10.1097/TA.0000000000002173, 30601456 PMC6682404

[166] LaGrone LN Stein D Cribari C Kaups K Harris C Miller AN . American association for the surgery of trauma/American College of Surgeons Committee on trauma: clinical protocol for damage-control resuscitation for the adult trauma patient. J Trauma Acute Care Surg. (2024) 96:510–20. doi: 10.1097/TA.0000000000004088 37697470

[167] Moren AM Hamptom D Diggs B Kiraly L Fox EE Holcomb JB . Recursive partitioning identifies greater than 4 U of packed red blood cells per hour as an improved massive transfusion definition. J Trauma Acute Care Surg. (2015) 79:920–4. doi: 10.1097/TA.0000000000000830, 26680135 PMC4778543

[168] Thomas SJ Patel VS Schmitt CP Zielinski AT Aboukhaled MN Steinberg CA . The effect of heterogeneous definitions of massive transfusion on using blood component thresholds to predict futility in severely bleeding trauma patients. J Clin Med. (2025) 14:5426. doi: 10.3390/jcm14155426, 40807047 PMC12347820

[169] Moore EE Moore HB Kornblith LZ Neal MD Hoffman M Mutch NJ . Trauma-induced coagulopathy. Nat Rev Dis Primers. (2021) 7:30. doi: 10.1038/s41572-021-00264-3, 33927200 PMC9107773

[170] Siegel JH Rivkind AI Dalal S Goodarzi S. Early physiologic predictors of injury severity and death in blunt multiple trauma. Arch Surg. (1990) 125:498–508. doi: 10.1001/archsurg.1990.01410160084019, 2322117

[171] Siegel JH. The effect of associated injuries, blood loss, and oxygen debt on death and disability in blunt traumatic brain injury: the need for early physiologic predictors of severity. J Neurotrauma. (1995) 12:579–90. doi: 10.1089/neu.1995.12.579, 8683609

[172] Kotwica Z Jakubowski JK. Head-injured adult patients with Gcs of 3 on admission--who have a chance to survive? Acta Neurochir. (1995) 133:56–9. doi: 10.1007/bf01404948, 8561037

[173] Majdan M Steyerberg EW Nieboer D Mauritz W Rusnak M Lingsma HF. Glasgow coma scale motor score and pupillary reaction to predict six-month mortality in patients with traumatic brain injury: comparison of field and admission assessment. J Neurotrauma. (2015) 32:101–8. doi: 10.1089/neu.2014.3438, 25227136 PMC4291088

[174] Tenovuo O Diaz-Arrastia R Goldstein LE Sharp DJ van der Naalt J Zasler ND. Assessing the severity of traumatic brain injury-time for a change? J Clin Med. (2021) 10:148. doi: 10.3390/jcm10010148, 33406786 PMC7795933

[175] Tien HC Cunha JR Wu SN Chughtai T Tremblay LN Brenneman FD . Do trauma patients with a Glasgow coma scale score of 3 and bilateral fixed and dilated pupils have any chance of survival? J Trauma. (2006) 60:274–8. doi: 10.1097/01.ta.0000197177.13379.f4 16508482

[176] Perel P Al-Shahi Salman R Kawahara T Morris Z Prieto-Merino D Roberts I . Crash-2 (clinical randomisation of an Antifibrinolytic in significant Haemorrhage) intracranial bleeding study: the effect of tranexamic acid in traumatic brain injury--a nested randomised, placebo-controlled trial. Health Technol Assess. (2012) 16:iii–xii, 1–54. doi: 10.3310/hta16130, 22417901

[177] Kockelmann F Maegele M. Acute Haemostatic depletion and failure in patients with traumatic brain injury (Tbi): pathophysiological and clinical considerations. J Clin Med. (2023) 12:2809. doi: 10.3390/jcm12082809, 37109145 PMC10143480

[178] Maegele M Aversa J Marsee MK McCauley R Chitta SH Vyakaranam S . Changes in coagulation following brain injury. Semin Thromb Hemost. (2020) 46:155–66. doi: 10.1055/s-0040-1702178, 32160642

[179] Barbosa RR Rowell SE Sambasivan CN Diggs BS Spinella PC Schreiber MA A predictive model for mortality in massively transfused trauma patients. J Trauma (2011) 71:S370–4. doi: 10.1097/TA.0b013e318227f18f. 21814106

[180] Mitra B Singh B Mathew J Stewart C Koolstra C Hendel S . Timing and volume of transfusion for adult major trauma patients with hemorrhagic shock: a registry-based cohort study. Trauma Surg Acute Care Open. (2024) 9:e001248. doi: 10.1136/tsaco-2023-001248, 38347897 PMC10860119

[181] Nunns GR Moore EE Stettler GR Moore HB Ghasabyan A Cohen M . Empiric transfusion strategies during life-threatening hemorrhage. Surgery. (2018) 164:306–11. doi: 10.1016/j.surg.2018.02.024, 29709368 PMC6056322

[182] Rubin M. Should we offer blood transfusions as a palliative therapy? Am J Bioeth. (2016) 16:62–4. doi: 10.1080/15265161.2016.1180444, 27292858

